# Presence of Nematodes, Mercury Concentrations, and Liver Pathology in Carnivorous Freshwater Fish from La Mojana, Sucre, Colombia: Assessing Fish Health and Potential Human Health Risks

**DOI:** 10.1007/s00244-025-01117-w

**Published:** 2025-02-20

**Authors:** Katerin Fuentes-Lopez, Jesus Olivero-Verbel, Karina Caballero-Gallardo

**Affiliations:** 1 Environmental and Computational Chemistry Group, School of Pharmaceutical Sciences, Zaragocilla Campus, University of Cartagena, 130014 Cartagena, Colombia; 2 Functional Toxicology Group, School of Pharmaceutical Sciences, Zaragocilla Campus, University of Cartagena, 130014 Cartagena, Colombia

## Abstract

Fish provide high-quality protein and essential nutrients. However, environmental pollution can lead to the accumulation of toxic substances such as mercury (Hg), with known negative impacts on human consumers. The aim of this study was to assess fish health and potential human health risks by analyzing the presence of nematodes, Hg concentrations, and liver pathology in freshwater fish from La Mojana, Colombia. For this purpose, 326 specimens were collected. Parasitic infection was evaluated using parasitological indices. Total Hg (T-Hg) was quantified using a direct mercury analyzer. Liver pathology was assessed through histopathological examination. Risk-based fish consumption limits were established by calculating the hazard quotient (HQ) and safe consumption limits. The overall prevalence and abundance of nematodes (*Contracaecum* sp.) were 46% and 2.72 ± 0.47 parasites/fish, respectively. *Sternopygus macrurus* exhibited the highest prevalence (100%) and parasite abundance (13.5 parasites/fish) during the rainy season, whereas *Hoplias malabaricus* demonstrated the greatest prevalence (100%) and abundance (14.8 parasites/fish) in the dry season. The average T-Hg was 0.31 ± 0.01 µg/g ww. During the rainy season, *S. macrurus* had the highest T-Hg levels (0.46 ± 0.08 µg/g ww); in the dry season, *Cynopotamus magdalenae* showed the highest T-Hg concentration (0.54 ± 0.03 µg/g ww). Significant positive relationships were recorded between T-Hg and parasite abundance, while these were negative with the condition factor. All specimens exhibited hepatic alterations. The HQ and Hg values suggest potential health risks from frequent fish consumption, especially in fish-dependent communities. These findings highlight the need for precautionary measures by health and environmental authorities to safeguard human and ecosystem health.

**Supplementary Information:**

The online version contains supplementary material available at 10.1007/s00244-025-01117-w.

Aquatic ecosystem pollution, driven by diverse anthropogenic activities, remains a major threat to both human health and environmental integrity. Heavy metals, due to their persistence, bioaccumulation, and potential to disrupt biological processes, are among the most concerning pollutants. Although health concerns regarding heavy metals, including mercury (Hg), have been recognized for decades (Kurland et al. 1960; UNEP 2018), recent studies have provided further evidence of additional risks and mechanisms of interference in aquatic organisms (Wu et al. 2024; Zhou et al. 2024; Erdemir et al. 2025). Metals can adhere to organic particles, settle in sediments, and enter the food web through bioaccumulation and biomagnification, either via sediment-dietary exposure or direct bioconcentration from water, leading to progressive accumulation along the trophic chain (Goretti et al. 2016; Pandiyan et al. 2021; Haynes and Zhou 2022). This accumulation results in various toxic effects, including genotoxic, physiological, embryotoxic, and immunotoxic alterations. Among these metals, Hg, particularly methylmercury (MeHg), stands out as a potent environmental stressor (Olivero-Verbel and Caballero-Gallardo 2013).

Widely distributed in our environment, Hg originates both from natural sources, such as volcanic eruptions, forest fires, biomass burning, and low-temperature volatilization, and from human activities, including coal combustion, metal ore smelting, waste management, and chemical production. These various sources contribute to the release of Hg into the atmosphere (Luo et al. 2020), where it can circulate for extended periods before being deposited on the earth's surface through rainfall. Upon deposition, Hg undergoes oxidation to its inorganic form (Hg^2^⁺) and accumulates in aquatic environments, particularly in sediments. In these anoxic conditions, microorganisms can methylate Hg^2^⁺, producing MeHg, a highly toxic compound. MeHg enters the food chain through plankton, bioaccumulates in aquatic organisms, and biomagnifies as it ascends the trophic level (TL), ultimately reaching top predators, including humans. This process underscores the significant ecological and health risks associated with Hg pollution (Bosch et al. 2016; Zhao et al. 2024).

There is evidence that Hg may suppress the immune system and could interfere with the host’s defense capacity against infectious pathogens, including parasites (Ebers Smith et al. 2018; Sun et al. 2018; Banday et al. 2020). This process might contribute to an increased incidence of diseases in organisms such as fish, which are of high importance to human consumers (Sanchez‐Dardon et al. 1999; Jalčová and Dvorožňáková 2014; Banerjee et al. 2015; Begam and Sengupta 2015). Although some authors have identified a positive correlation between Hg concentration and parasite abundance in fish (Olivero-Verbel and Caballero-Gallardo 2013), the relationship can shift from negative to positive depending on contaminant levels, fish species, and developmental stage (Molbert et al. 2021).

In addition, parasites may vary in their capacity to sequester or influence the distribution of metals in host tissues. For instance, while certain parasites can accumulate metals, Hg often remains in fish tissues rather than in the parasites themselves (Brázová et al. 2024). Parasitism occurs frequently, rendering most fish susceptible to infection at various developmental stages, especially when exposed to organic contaminants and metals, which may result in oxidative stress (El-Hak et al. 2022), inflammatory lesions, immunosuppression, and physiological or endocrine alterations (Choudhury et al. 2021; Liu et al. 2023). Elevated contaminant concentrations can negatively affect both aquatic organisms and their associated parasites. Eutrophic conditions, characterized by excessive nutrient enrichment, can alter water quality and disrupt ecological balance, potentially triggering biomarker responses in fish, such as oxidative stress or enzymatic activity changes (Latief et al. 2023). The extent of bioaccumulation could also differ according to fish size, weight, lifestyle, and degree of infestation (Graci et al. 2017). These observations underscore the need for further parasitological research in metal-polluted habitats (Nur et al. 2020), as a more comprehensive understanding of host–parasite–contaminant interactions is crucial for evaluating both ecological and health-related implications (Graci et al. 2017).

Carnivorous fish are of particular interest because they may be the most susceptible to contamination. They are exposed not only to bioaccumulation processes that occur over time but also to biomagnification processes due to their consumption of other fish, which are likely to be significantly contaminated (Olivero-Verbel and Caballero-Gallardo 2013; Marrugo-Negrete et al. 2020).

Nematode parasites belonging to the Anisakidae family are the most common. The Anisakis genus has been widely referenced globally; however, in Colombia, nematodes of the genus *Contracaecum* have been recorded in different species of fish (Olivero-Verbel et al. 2011); in this way, this infection compromises not only health of the fish but the quality and food safety. The quality of the fish decreases immediately if they are infected with these parasites, which generates a high risk of human infection. This, in turn, could trigger a series of negative effects from their consumption, including zoonoses, allergies, anaphylactic reactions, and alterations at the central nervous system level (Olivero-Verbel et al. 2006).

*Contracaecum* nematodes, commonly found in the muscle and visceral organs of fish, can induce physiological stress, tissue damage, and immune suppression, compromising fish health and growth. In humans, consuming raw or undercooked fish containing infective larvae may result in anisakiasis, a gastrointestinal condition causing abdominal pain, nausea, and, in severe cases, intestinal complications. Even after proper cooking or freezing, allergenic excretory-secretory (ES) proteins from the parasite can persist, potentially triggering allergic reactions, including anaphylaxis (Carballeda-Sangiao et al. 2014; Gahoi et al. 2019).

In Colombia, more specifically in the department of Sucre, the La Mojana region is one of the richest areas in biodiversity, a food producer and abundant in water resources. However, it presents high rates of poverty and dependence on the exploitation of resources such as soil and water, since its main economic activities are agriculture and fishing (DNP 2015; Buelvas-Jimenez and González-Pedraza 2022; PNUD 2022; Caro et al. 2023). The impact caused by gold mining, ferronickel and coal exploitation activities, and the indiscriminate use of pesticides in agriculture results in the release of numerous pollutants into the atmosphere and soil, which eventually accumulate in water bodies (Morante and Negrete 2018) that reaches the La Mojana region through the Cauca, Magdalena and San Jorge rivers (Morante and Negrete 2018) and directly impacts the fish, thus representing a risk factor for the community.

La Mojana, Colombia, has been one of the areas most affected by gold mining, receiving a large amount of contaminants from these activities (Baleta-Anaya et al. 2022; Romero-Suárez et al. 2022). Mercury has been found in species of commercial interest in the region, especially carnivorous, such as Bagre rayado (*Pseudoplatystoma magdaleniatum*), Pacora (*Plagioscion surinamensis*), Mojarra amarilla (*Caquetaia kraussii*), Moncholo (*Hoplias malabaricus*) and Bagre blanquillo (*Sorubim cuspicaudus*) (Marrugo-Negrete et al. 2008). The high average consumption of these fish seems to activate several molecular mechanisms that can lead to carcinogenic and teratogenic processes (Galeano-Páez et al. 2021; Baleta-Anaya et al. 2022).

The fish species most consumed in this region are mainly those with carnivorous trophic ecology such as Bagre rayado (*P. magdaleniatum*), Bagre blanquillo (*S. cuspicaudus*), Doncella (*Ageneiosus pardalis*), Pacora (*P. surinamensis*), Moncholo (*H. malabaricus*), Mojarra Amarilla (*C. kraussii*), Barbul (*Pimelodus clarias*); in addition, there are omnivores such as Comelón (*Megaleporinus muyscorum*) and detritivores such as the Viejito (*Curimata magdalenae*) and Bocachico (*Prochilodus magdalenae*). Therefore, knowing the health status of these species is of great interest (Baleta-Anaya et al. 2022).

Both Hg contamination and parasitic infections are critical factors in evaluating fish health and quality, particularly in regions where fish is a primary food source. Reports of parasitic nematodes in Colombian freshwater fish are scarce, with even fewer studies focusing on species commonly consumed in La Mojana, Sucre. Investigating the presence of nematode infections in these species is essential, as they can impact fish health by causing physiological stress, tissue damage, and potential interactions with toxic elements such as Hg. Considering these risks, this study hypothesizes that nematode infections and mercury accumulation in carnivorous fish species from La Mojana may be linked to liver damage and could pose potential health risks to consumers. Therefore, the objective of this study was to assess fish health and potential human health risks by analyzing nematode infections, Hg concentrations, and liver pathology in carnivorous freshwater fish from La Mojana, Sucre, Colombia.

## Materials and Methods

### Study Area

**Fig. 1:**
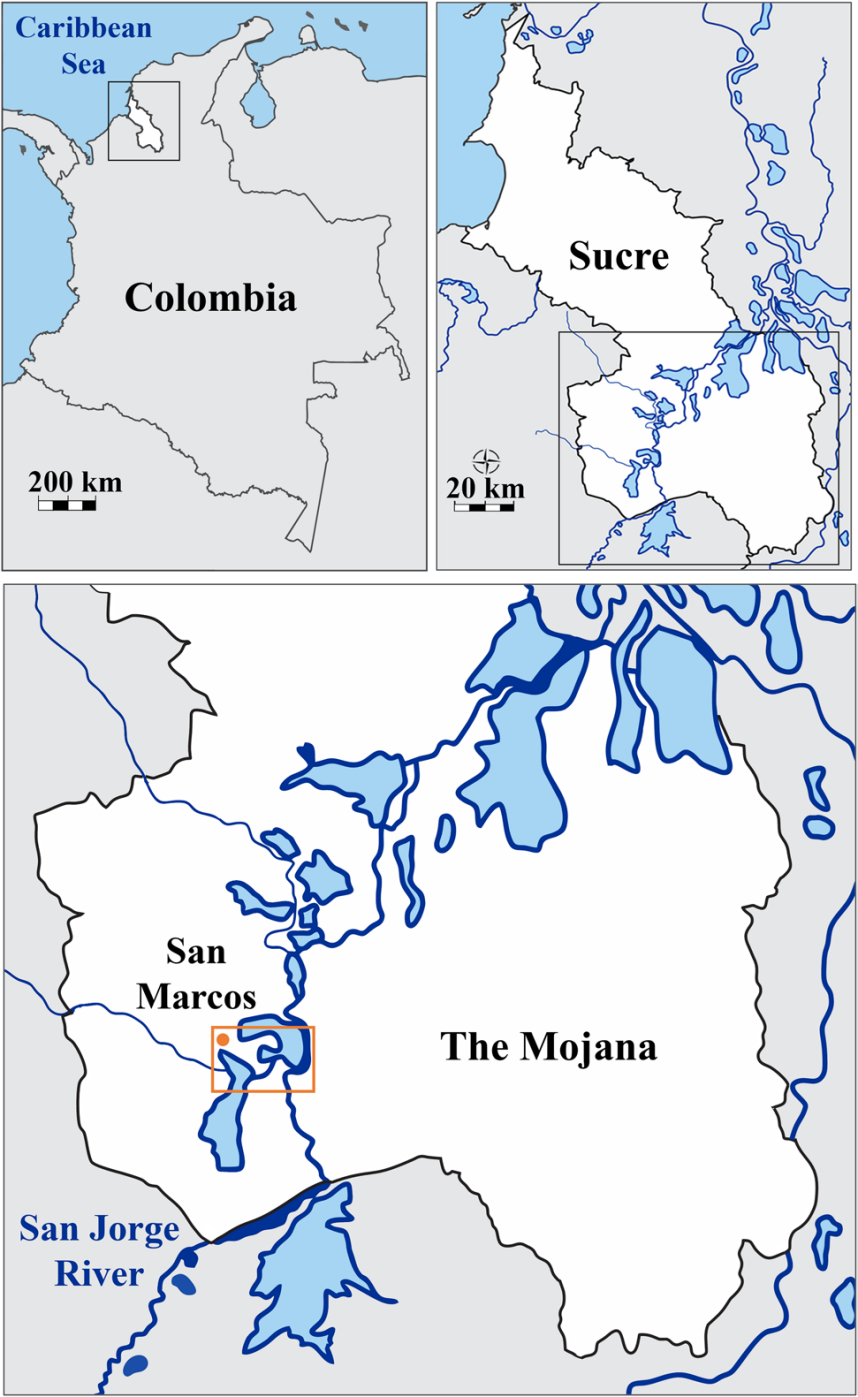
Map of study area indicating the geographic location of La Mojana (Sucre) and San Jorge River (Colombia). The orange box indicates the location of the sampling site

The research was carried out in the San Jorge River adjacent to the municipality of San Marcos (8° 35′ 06'' N, 75° 07′ 16.39'' W), situated south of the department of Sucre, serving as the gateway to the La Mojana region in northwestern Colombia (Baleta-Anaya et al. 2022). La Mojana has an approximate area of 5545 km^2^ (Marrugo-Negrete et al. 2018a). It has a humid and dry tropical climate, with temperatures ranging between 28 and 33 °C (82 and 95°F). Geographically delimited to the east by the Cauca River, to the west by the San Jorge River and the Ayapel swamp, to the northeast by the Loba branch of the Magdalena River, to the south by the highlands of Caucasia and the Ayapel mountain range (Fig. 1) (Díaz-Granados et al. 2001).

### Specimen Collection

A total of 326 specimens (nine fish species: *Pseudoplatystoma magdaleniatum,* *Hoplias malabaricus*, *Plagioscion surinamensis*, *Sorubim cuspicaudus*, *Cynopotamus magdalenae*, *Polydactylus virginicus*, *Trachelyopterus insignis*, *Caquetaia kraussii*, and *Sternopygus macrurus*) were collected in San Marcos, a San Jorge River basin area, state of Sucre (Colombia), between September 2022 and April 2023. Sampling was conducted during both the rainy (April to November) and dry (December to March) seasons. The seasonal classification was validated using a Walter-Lieth climatic diagram, constructed in R based on historical climate data for San Marcos, Sucre, from 1991 to 2021 (Climate-Data 2024). This approach ensured the alignment of the sampling period with the region's established climatic patterns (Fig. S1).

Fishermen from the municipality of San Marcos participated in the capture of the fishes, using cast nets and trammel nets. The fish were collected with a size of ≥ 17 cm (average capture size) and stored on ice until in situ processing (Olivero-Verbel et al. 2009). The TL was determined using the FishBase database, accessible at https://fishbase.se/search.php, which is a comprehensive resource containing trophic level information for numerous fish species. This information is typically derived from dietary data and ecological analyses, providing a reliable basis for understanding the feeding relationships and ecological roles of fish within aquatic ecosystems.

### Morphometric Analysis of Fish and Larvae Extraction

All specimens were carefully cleaned, and total fish length (L, cm) and weight (W, g) were recorded for each specimen. Each specimen was then dissected, and its internal organs were visually examined. Simultaneously, all detected parasite larvae were collected and counted for each specimen and subsequently fixed with buffered formalin. Additionally, the liver was removed, and its W recorded. Biometric parameters indicative of fish health status were then calculated. The Condition Factor (CF) (de Moraes Vazzoler, 1996) was determined to assess the robustness of each fish, using the formula CF = 100 × W/L^3^; and the hepatosomatic index (HSI) (Maddock and Burton 1998), measured as HSI = (LW/W) × 100, where LW is the liver weight (g) (Olivero-Verbel et al. 2009, 2011).

### Parasitological Indices and Morphological Identification

The prevalence, abundance and mean parasite intensity were assessed in the total number of collected fish, following the criteria of Bush et al. (1997) and Margolis et al. (1982). Preliminary morphological identification of the parasites was based on the keys of Anderson (2000).

### Molecular Characterization of Nematode and Fish Species by Sequencing

Parasites stored at -20 °C, their DNA was extracted from 12 larvae with two replicates each (Umehara et al. 2007) using the PureLink® Genomic DNA Mini Kit Thermo Fisher (Invitrogen, Thermo Fisher Scientific, Waltham, MA, USA) following the manufacturer's recommendations. DNA integrity was verified by 1% agarose gel electrophoresis and purity using the Nanodrop 2000 spectrophotometer. Genetic identification was performed by conventional PCR using Veriti 96-Well Fast Thermal Cycler (Applied Biosystems™, Foster City, CA, USA) with primers used for the identification of *Contracaecum* sp. nematodes (Nematoda: Anisakidae): rrnS (small subunit of mitochondrial ribosomal RNA) (Pekmezci and Yardimci 2019; Carmeno et al. 2022) (Table S1). The amplification conditions for molecular markers of rrnS were established using the modified protocols of D'Amelio et al. (2000) and D'Amelio et al. (2007). PCRs were carried out in a 25 µL reaction volume, containing 5 μL DNA (10 ng/µL), 1 µL of each primer 10 mM, 1 μL of GC enhancer and 12.5 μL of AmpliTaq Gold™ 360 Master Mix (Thermo Fisher Scientific, Waltham, MA, USA). The amplification protocol was performed using the following conditions: initial denaturation at 95 °C for 10 min, followed by 35 cycles at 95 °C for 15 s (denaturation), at 52.4 °C for 30 s (annealing), at 72 °C for 1 min (extension), and a final extension at 72 °C for 7 min (Vipindas et al. 2020).

### PCR Product Purification, Sequencing and Phylogenetic Inference

The PCR products amplified for each gene evaluated were purified using the QIAquick PCR Purification Kit (Qiagen, CA, USA), following the manufacturer's recommendations, and subsequently used as templates for the sequencing reactions. DNA sequencing was performed through the Sequencing and Molecular Analysis Service (SSiGMol) (http://www.ssigmol.unal.edu.co/), Institute of Genetics, National University of Colombia, using an automated capillary electrophoresis system (Applied Biosystems 3130xl Genetic Analyzer) with the BigDye Terminator v3.1 Cycle Sequencing Kit (Applied Biosystems). The obtained sequences were then aligned using the Clustal W package within MEGA software version 11. The consensus sequences or contigs resulting from the sequence alignment were compared with other similar sequences using BLASTn (Basic Local Alignment Search Tool), as reported in previous studies (Thompson et al. 1997; Jiang et al. 2010). Finally, the subsequent analysis involved the construction of phylogenetic trees using the Neighbor-Joining (NJ) method (Kumar et al. 2004; Jiang et al. 2010), incorporating a bootstrapping confidence test of 1000 replicates (Jiang et al. 2010). Evolutionary distances were calculated using the Jukes–Cantor method (Huelsenbeck 1995; Kumar et al. 2004), and the resulting phylogenetic tree was represented by a cladogram.

### Total Hg (T-Hg) Analysis and Quality Control

The T-Hg analysis was conducted following the procedures outlined in previously published works (Olivero-Verbel et al. 2015; Palacios-Torres et al. 2018; Carranza-Lopez et al. 2019; Caballero-Gallardo et al. 2022). To minimize potential alterations in T-Hg concentration, 10 mg of fresh fish muscle were placed in a sample holder and directly analyzed using a direct mercury analyzer (DMA-80 TriCell Milestone; Milestone, Italy), as this method allows for accurate quantification without the need for sample drying, which can affect Hg content. T-Hg quantification was performed using calibration curves prepared with the Certified Reference Material (CRM) for fish muscle (ERM-BB422, reference value: 0.601 µg/g). The calibration curves consisted of at least five points with a regression coefficient (R^2^ ≥ 0.999), following USEPA method 7473. The detection limit (LOD, Limit of Detection) was calculated as three times the standard deviation of the concentration obtained from empty boats (0.003 μg/g). Precision was calculated as the coefficient of variation between replicates of the same sample (< 10% RSD, Relative Standard Deviation). Each sample was analyzed in duplicate. Accuracy was verified by testing CRM DORM-3 from the National Research Council of Canada (reference value, 0.382 μg/g dw; obtained value (n = 5), 0.379 ± 0.007 μg/g dw; recovery percentage, 99.8%). At least one method blank (empty preheated combustion boat) and one CRM were tested on every 10 samples.

### Histological Analysis

Samples stored in 10% buffered formalin were dehydrated in increasing concentrations of ethyl alcohol (70%—96%). Subsequently, they were embedded in paraffin to make 5 µm-thick histological sections, stained with hematoxylin–eosin (H&E), and then examined under the Leica DM2500 optical microscope, DFC550 camera, and Leica Application Suite V4 software with 10X and 40X objectives. The Nis-Elements version 3.0 software (Ameur et al. 2012; Caballero-Gallardo and Olivero-Verbel 2016) was used for photographic registration. Liver samples were randomly selected from the fish collected in both the rainy and dry seasons using a random sampling script in R. A total of four liver samples per species were selected for histological analysis. This approach ensured an unbiased representation of fish health across species and seasons while considering financial and logistical constraints. The selection process excluded empty data and avoided repetition within the same species. Tissue alterations found in each studied organ were recorded, specifically histopathological findings at the hepatic parenchyma level (steatosis, inflammatory lymphocytic infiltrates, inflammation with melanomacrophages, necrosis, apoptosis, hepatic congestion, blood congestion, hepatocyte and nuclear hypertrophy, parasite cysts, among others) (Hadi and Alwan 2012; Palacios et al. 2015; Pastor et al. 2015; Dang et al. 2017; Leone et al. 2018). Each finding was semi-quantitatively assessed using a range of occurrence (Muznebin et al. 2023). This system categorizes the severity of alterations based on the extent and intensity of tissue damage observed in the hepatic parenchyma. The scoring criteria used were: 0 = absent, 1 = mild, 2 = moderate, and 3 = severe. Each liver sample was evaluated for the presence of histopathological alterations such as steatosis, inflammatory lymphocytic infiltrates, melanomacrophage centers, necrosis, apoptosis, hepatic congestion, blood congestion, hepatocyte and nuclear hypertrophy, and parasite cysts. To visualize the severities of liver alterations in fish, a heatmap was generated by clustering liver histopathology data for all species based on Euclidean distance. This was accomplished using the heatmap.2 function from the gplots package in R version 4.3.1. The color palette represented four severity levels: absent (0.0–0.4), mild (0.5–1.4), moderate (1.5–2.4), and severe (2.5 to > 3.0).

### Risk-Based Fish Consumption Limits

To assess the potential health risks associated with Hg exposure through fish consumption, the hazard quotient (HQ) was calculated following the guidelines established by the US Environmental Protection Agency (USEPA 2000). The HQ was determined using the formula HQ = EDI/RfD, where the Estimated Daily Intake (EDI) represents the level of Hg exposure, and RfD is the Reference Dose, set at 0.1 μg/kg/day. The EDI was calculated using the equation: EDI = C × MS/W, where C represents the mean MeHg concentration in fish muscle, MS corresponds to the standard portion size (230 g for an average adult), and W is the standard body weight (70 kg). Since approximately 90% of the T-Hg present in fish muscle exists in the form of MeHg, C was adjusted using the factor C = 0.9 × T-Hg (USEPA 1999). An HQ value below 1 indicates minimal risk of adverse health effects in vulnerable populations, whereas an HQ greater than 1 suggests that non-cancerous systemic effects may occur, particularly for sensitive groups such as pregnant women and children (Gutiérrez-Mosquera et al. 2021). The evaluated portions were 113 g for pregnant/lactating women and 31.2 g, 42.5 g, and 48.2 g for children aged 3, 4, and 5 years, respectively, with corresponding standard body weights of 75 kg, 13.4 kg, 15.7 kg, and 21.1 kg (USEPA-FDA 2018; Caballero-Gallardo et al. 2022). Finally, the maximum allowable weekly fish consumption rate was estimated using the formula CRmw = 49/(C × MS), a standard approach for assessing non-cancer health effects without expected chronic systemic toxicity (Olivero-Verbel et al. 2015, 2016; Palacios-Torres et al. 2020).

### Data Analysis

Data were presented as mean ± standard error. Normality and homogeneity of variance were assessed using Kolmogorov–Smirnov and Bartlett tests, respectively. When data did not meet the assumptions of normality or homogeneity, nonparametric tests were applied. Student's t test was used to compare T-Hg concentrations within species across seasons, while the Mann–Whitney U test was used when assumptions of normality were not met. ANOVA and Kruskal–Wallis test assessed T-Hg differences between species and parasite abundance among infected species. These tests were conducted separately by season, with species analyzed individually for comparisons and grouped for overall trends in parasite abundance and contamination levels. Fisher’s exact test compared prevalence across seasons. Spearman correlations explored associations among quantitative variables. Principal Component Analysis (PCA) was employed to reduce dimensionality and summarize the dataset’s variability while visualizing relationships among multiple variables. Finally, multiple linear regression analysis was used to assess the influence of CF, Hg, and TL on parasite abundance, with the Akaike Information Criterion (AIC) applied to identify the most informative model. All analyses were performed using GraphPad Prism 9 and the R software (Version 4.3.1), with statistical significance set at *p* < 0.05.

## Results

### Morphometric Analysis of Fish and Larvae Extraction

A total of 326 fish representing nine carnivorous species (TL 3.2–4.5) were collected during both rainy and dry seasons in the San Jorge River (Table 1). Body size ranged from 17.55 ± 0.26 cm to 82.17 ± 3.67 cm for *Trachelyopterus insignis* (rainy season) and *Sternopygus macrurus* (rainy), respectively. HSI values were highest in *T. insignis* and lowest in *P. virginicus* or *S. macrurus*, depending on the season. Condition factor was highest in *C. kraussii* and lowest in *S. macrurus* during both seasons. These morphometric and physiological variations were evaluated alongside seasonal data to ensure a comprehensive analysis of fish health and ecological dynamics under fluctuating environmental conditions.

**Table 1:** Trophic level and morphometric/biometric parameters of fish samples from the San Jorge River

Common name	Scientific name	Trophic level	Season	n	Total length (cm)	Total weight (g)	Condition factor	Hepatosomatic index (%)
Moncholo	*Hoplias malabaricus* (Bloch, 1794)	4.5	Rainy	17	30.8 ± 0.73	331.2 ± 24.39	1.10 ± 0.02	1.47 ± 0.14
			Dry	17	28.6 ± 0.74	232.6 ± 18.23	0.96 ± 0.01	1.26 ± 0.07
Pacora	*Plagioscion surinamensis* (Bleeker, 1873)	4.5	Rainy	23	28.8 ± 053	183.3 ± 9.89	0.75 ± 0.02	0.74 ± 0.05
			Dry	38	31.2 ± 5.54	159.4 ± 14.47	0.86 ± 0.04	1.06 ± 0.05
Bagre rayado	*Pseudoplatystoma magdaleniatum* (Buitrago-Suárez & Burr, 2007)	4.3	Rainy	5	48.3 ± 3.87	747.2 ± 196.15	0.59 ± 0.04	1.17 ± 0.12
			Dry	16	41.9 ± 0.88	375.0 ± 31.72	0.50 ± 0.01	0.93 ± 0.04
Bagre blanquillo	*Sorubim cuspicaudus* (Littmann, Burr & Nass, 2000)	4.3	Rainy	10	46.6 ± 1.93	488.5 ± 7.36	0.47 ± 0.03	0.99 ± 0.09
			Dry	19	59.5 ± 15.41	437.3 ± 9.46	0.43 ± 0.04	1.09 ± 0.10
Chango	*Cynopotamus magdalenae* (Steindachner, 1879)	4.2	Rainy	34	27.7 ± 0.96	187.5 ± 1.57	0.91 ± 0.03	0.73 ± 0.03
			Dry	29	23.6 ± 0.58	160.3 ± 3.08	1.11 ± 0.11	1.09 ± 0.06
Barbul	*Polydactylus virginicus* (Linnaeus, 1758)	3.7	Rainy	12	24.1 ± 0.54	132.0 ± 9.87	0.93 ± 0.01	0.49 ± 0.04
			Dry	21	21.6 ± 0.59	83.9 ± 7.94	0.79 ± 0.02	1.07 ± 0.08
Cachegua	*Trachelyopterus insignis* (Steindachner, 1878)	3.5	Rainy	3	20.6 ± 1.62	111.3 ± 21.67	1.25 ± 0.07	1.79 ± 0.19
			Dry	12	20.5 ± 0.72	119.2 ± 15.89	1.30 ± 0.05	1.73 ± 0.13
Mojarra amarilla	*Caquetaia kraussii* (Steindachner, 1878)	3.4	Rainy	18	21.17 ± 0.45	150.28 ± 9.70	1.57 ± 0.05	0.89 ± 0.06
			Dry	22	17.55 ± 0.26	72.5 ± 4.68	1.31 ± 0.04	0.83 ± 0.15
Mayupa	*Sternopygus macrurus* (Bloch & Schneider, 1801)	3.2	Rainy	6	82.17 ± 3.67	991.0 ± 140.75	0.17 ± 0.01	0.70 ± 0.06
			Dry	24	63.53 ± 2.13	522.8 ± 52.45	0.19 ± 0.01	0.65 ± 0.04

Trophic levels for fish species were obtained from FishBase (https://www.fishbase.se); n, Sample size

### Parasitological Indices and Morphological Identification

**Fig. 2:**
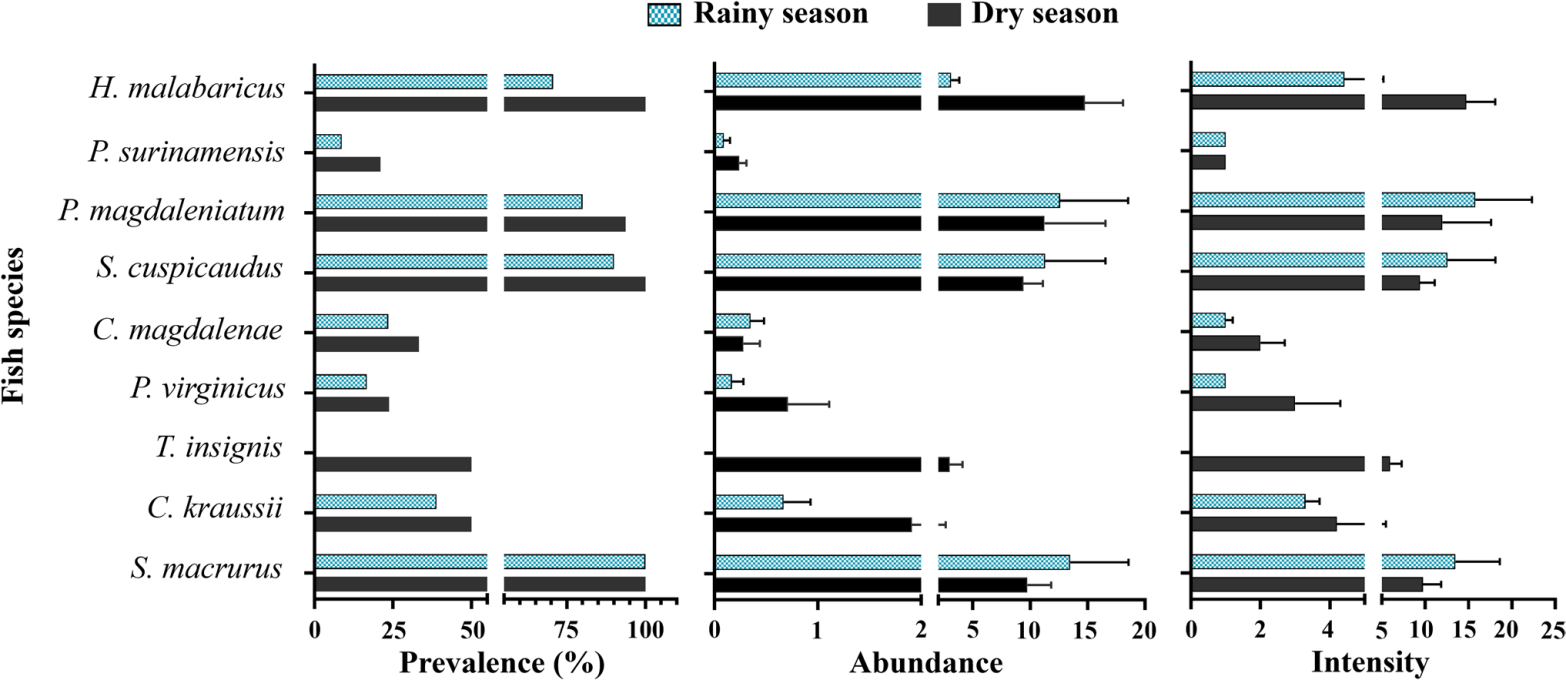
Nematode prevalence, abundance, and intensity in fish species from La Mojana Region, Sucre

The prevalence, abundance, and parasite intensity are depicted in Fig. 2. A total of 1291 *Contracaecum* nematodes were collected from 326 fish sampled from the San Jorge River, with an overall prevalence of 46% and abundance of 2.72 ± 0.47 parasites/fish. These *Contracaecum* nematodes measure an average of 16.2 mm (range 5–28 mm). In the rainy season, the prevalence of nematodes was 39.0%, with average abundance and intensity of 2.64 ± 0.64 and 6.76 ± 1.02 nematodes/fish, respectively. In the dry season, the prevalence was 55%, with average abundance and intensity of 4.81 ± 0.70 and 8.82 ± 1.14 nematodes per fish, respectively.

The species exhibiting the highest rates of prevalence and abundance during the rainy season are *S. cuspicaudus* (90% and 11.30) and *S. macrurus* (100% and 13.50), in contrast to *T. insignis* (0% and 0) and *P. surinamensis* (8.70% and 0.09), which show the lowest prevalence and abundance. In the dry season, four species are factored into the equation, surpassing the 90% parasitic prevalence threshold; consequently, the highest prevalences and abundances are observed in the species *H. malabaricus* (100% and 14.76), *P. magdaleniatum* (93.75% and 11.25), *S. cuspicaudus* (100% and 9.42), and *S. macrurus* (100% and 9.75).

The statistical analysis using Fisher's exact test revealed a significant association between parasitic prevalence and seasonal variation among the studied fish species. Specifically, *H. malabaricus* (*p* < 0.001), *P. magdaleniatum* (*p* = 0.0054), *P. surinamensis* (*p* = 0.0281), *S. cuspicaudus* (*p* = 0.0015), and *T. insignis* (*p* < 0.001) exhibited significantly different infection rates between the rainy and dry seasons. No significant relationships were observed for the remaining species.

**Fig. 3:**
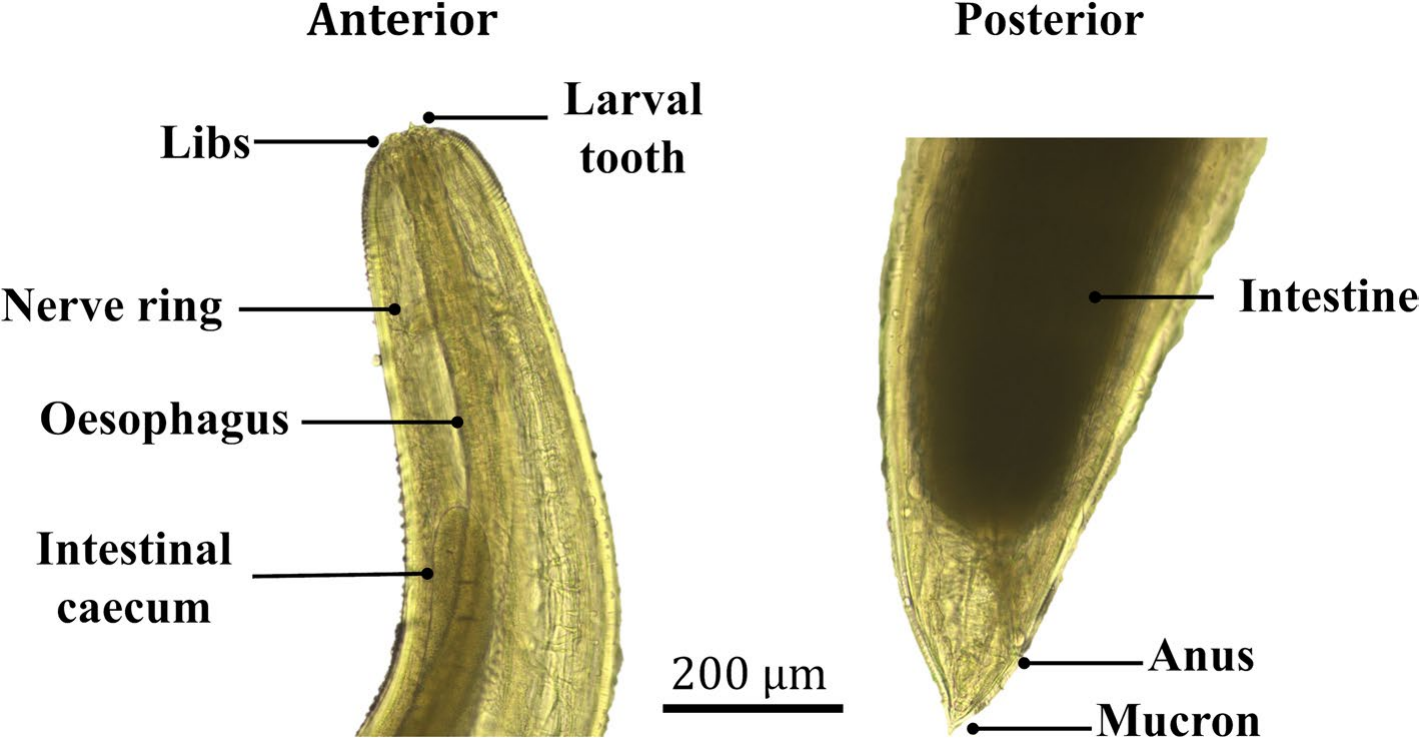
Morphological characteristics of the nematode found in *H. malabaricus* from La Mojana, Colombia

The morphological identification of the nematodes found in the fish (Fig. S2) showed some typical and specific characteristics of the *Contracaecum* species, of the Anisakidae family, as shown in Fig. 3, which include the presence of a prominent intestinal caecum that extends from the ventricle to the vicinity of the nerve ring, and a ventricular appendage facing the hindlimb. In addition, it has lips with a cuticular tooth between them. The nematodes were found mostly in the intestinal mesentery, as well as inside the muscle. Other characteristics of the nematodes found in the infected species were whitish color with transversely striated cuticle; rounded head with three lips, an esophagus, an intestinal caecum and a nerve ring found in the anterior third of the parasite, which are typical of the third larval instar (L3) of *Contracaecum* sp.

### Molecular Characterization of Nematode and Fish Species by Sequencing

Based on the 485 bp sequences obtained, the blast analysis of the rrnS gene region from the DNA samples of 12 *Contracaecum* sp. larvae revealed a 94.30% identity with sequences of *Contracaecum microcephalum* voucher Php1 12S small subunit ribosomal RNA gene, partial sequence; mitochondrial, previously deposited in GenBank under accession numbers EF014282.1. This high level of similarity suggests a taxonomic affiliation with *C. microcephalum* for the sequences obtained in this study. Additionally, *C. rudolphii* C D'Amelio et al. 2007 isolate CrCC75 small subunit ribosomal RNA gene, partial sequence; mitochondrial, was identified with an 92.75% identity and accession code FJ426248.1.

**Fig. 4:**
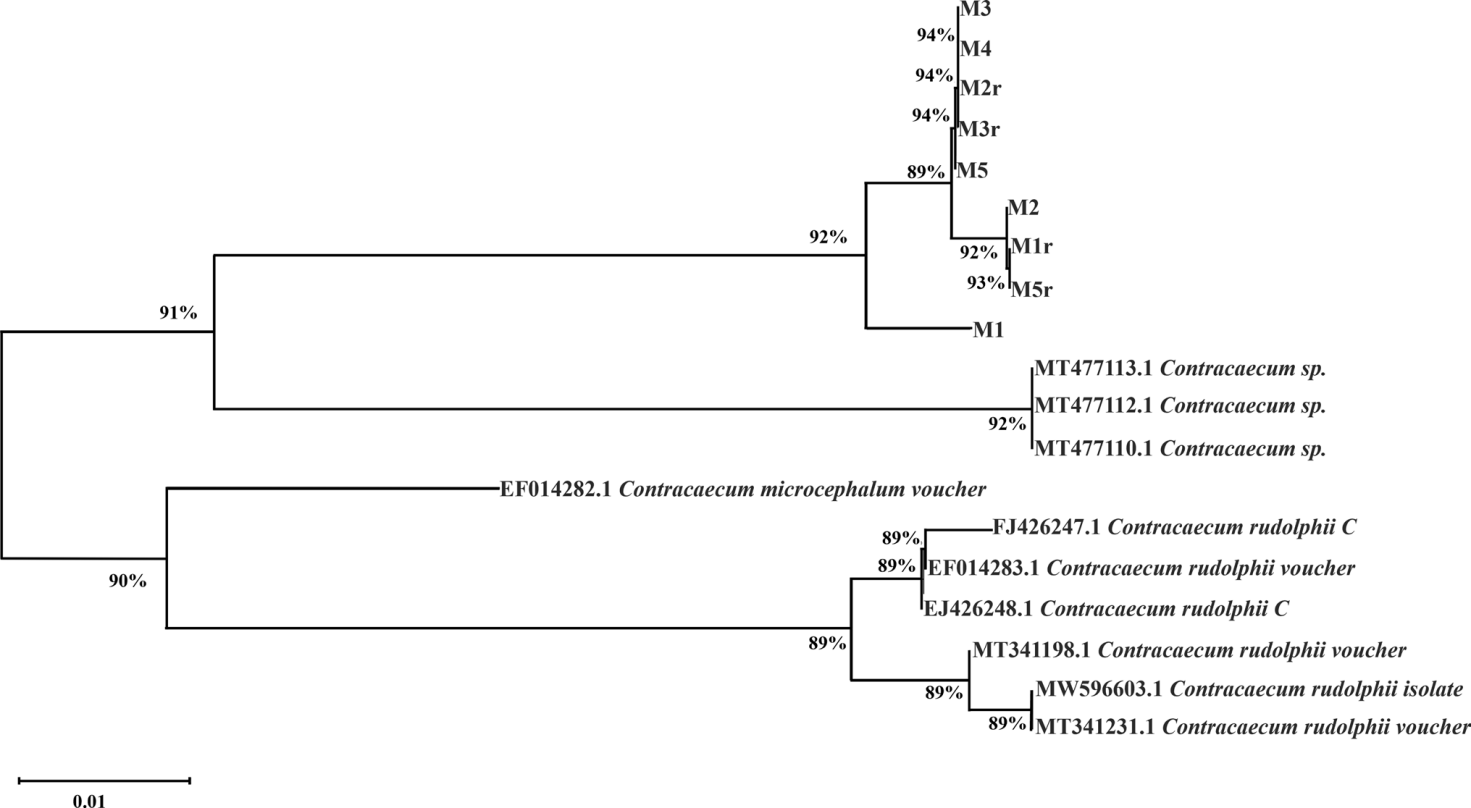
Phylogenetic tree of species of the superfamily Contracaecum and the parasite studied based on the RRNS sequence. The evolutionary history was deduced using the Neighbor-Joining method. The percentages of Bootstrap (1000 bootstrap replicates) are shown next to the branches. M1-M6 represent the analyzed samples

A phylogenetic tree was constructed with the species that were genetically most closely related to the consensus sequences of the parasite studied, these being those belonging to the superfamily *Contracaecum* (Fig. 4).

### T-Hg Concentrations and Seasonal Comparisons

**Fig. 5:**
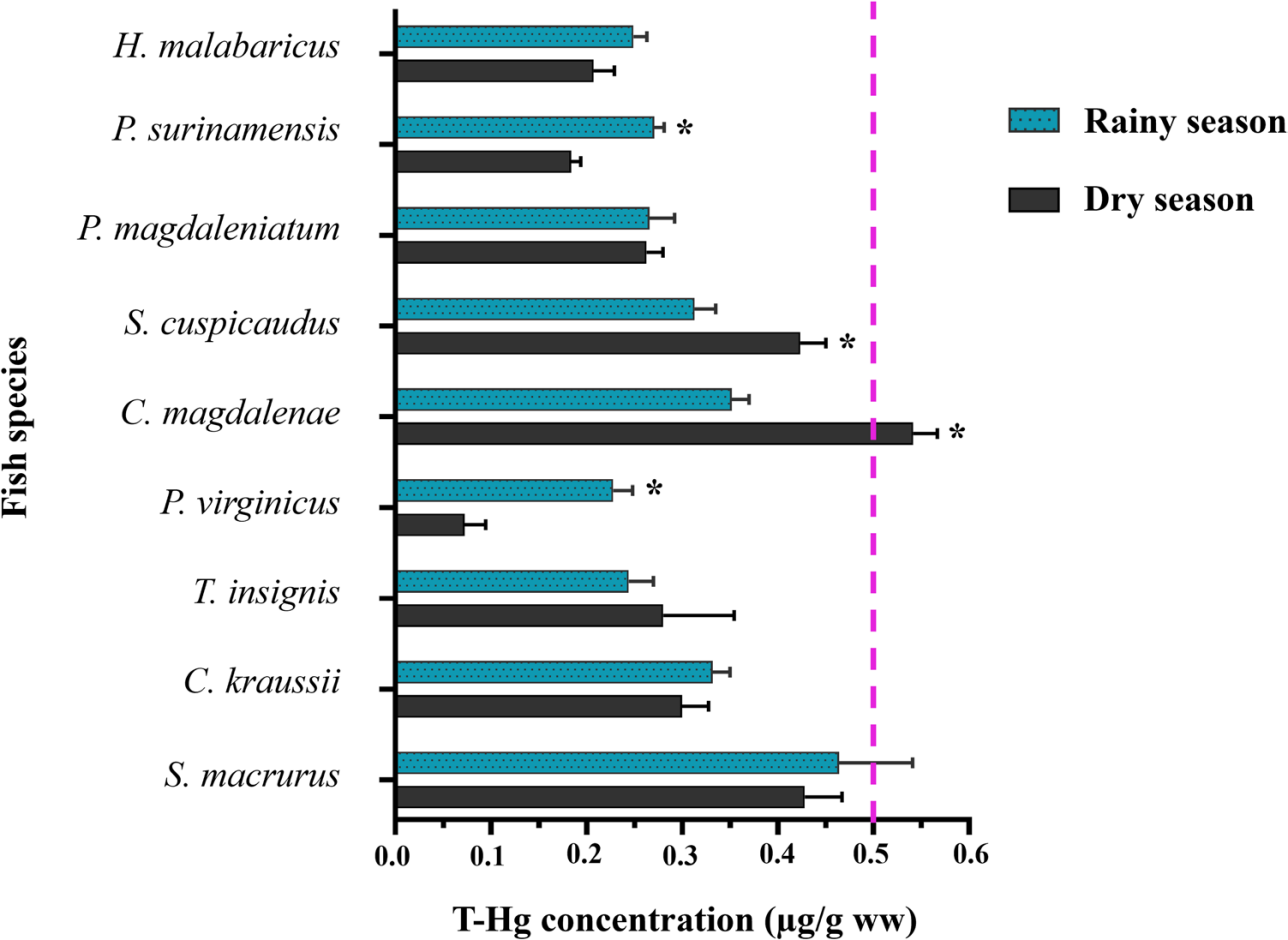
T-Hg concentration in fish species from La Mojana Region, Sucre. *Significant differences (*p* value < 0.05)

The concentration of T-Hg in evaluated fish is presented in Fig. 5. The average T-Hg concentration in analyzed fish was 0.31 ± 0.01 µg/g ww (range 0.01–0.85 µg/g ww). Hg concentrations in various fish species during the rainy and dry seasons revealed notable patterns in Hg contamination within the aquatic ecosystem. In the rainy season, greater concentrations were prominent in *S. macrurus* (0.46 ± 0.08 µg/g ww), *C. magdalenae* (0.35 ± 0.02 µg/g ww), and *S. cuspicaudus* (0.31 ± 0.02 µg/g ww). During the dry season, *C. magdalenae* had the highest T-Hg concentration (0.54 ± 0.03 µg/g ww), followed by *S. cuspicaudus* (0.42 ± 0.03 µg/g ww) and *S. macrurus* (0.43 ± 0.04 µg/g ww), while *P. virginicus* presented the lowest concentration (0.07 ± 0.02 µg/g ww) (Table S2).

The seasonal comparison using the Mann–Whitney test (*U* = 11,672, *p* = 0.229) revealed no statistically significant differences in overall T-Hg concentrations between seasons. However, species-specific variations in T-Hg concentrations were observed, indicating distinct patterns among the investigated fish species rather than uniform seasonal trends (Fig. S3). Significant differences were observed between rainy and dry seasons in the species *P. surinamensis*, *S. cuspicaudus*, *C. magdalenae*, and *P. virginicus* (Table S2). These results highlight the seasonal influence on Hg concentrations in different fish species, emphasizing the importance of considering temporal variations when assessing pollution in aquatic ecosystems and its implications for environmental health. Further analysis revealed significant variations in T-Hg levels between the rainy and dry seasons across individual species. *P. surinamensis* and *S. macrurus* exhibited higher concentrations of T-Hg during the rainy season (0.27 ± 0.01 and 0.23 ± 0.02 µg/g ww, respectively) compared to the dry season (0.18 ± 0.01 and 0.07 ± 0.02 µg/g ww), indicating a significant difference (*P. surinamensis*: t = 5.559, *p* < 0.001; *S. macrurus*: 4.693, *p* < 0.001, respectively). In contrast, *C. magdalenae* displayed higher T-Hg levels during the dry season (0.54 ± 0.03 µg/g ww) than during the rainy season (0.35 ± 0.02 µg/g ww), also with a significant difference (t = 6.206, *p* < 0.001). *S. cuspicaudus* showed similar patterns, with higher T-Hg concentrations during the dry season (0.42 ± 0.03 µg/g ww) compared to the rainy season (0.31 ± 0.02 µg/g ww), demonstrating highly significant differences (t = 2.739, *p* = 0.011). These findings suggest that Hg accumulation patterns vary significantly between species and seasons, reflecting a complex interaction of environmental and biological factors influencing Hg distribution in aquatic ecosystems.

### Correlation Analysis Between Variables

**Fig. 6:**
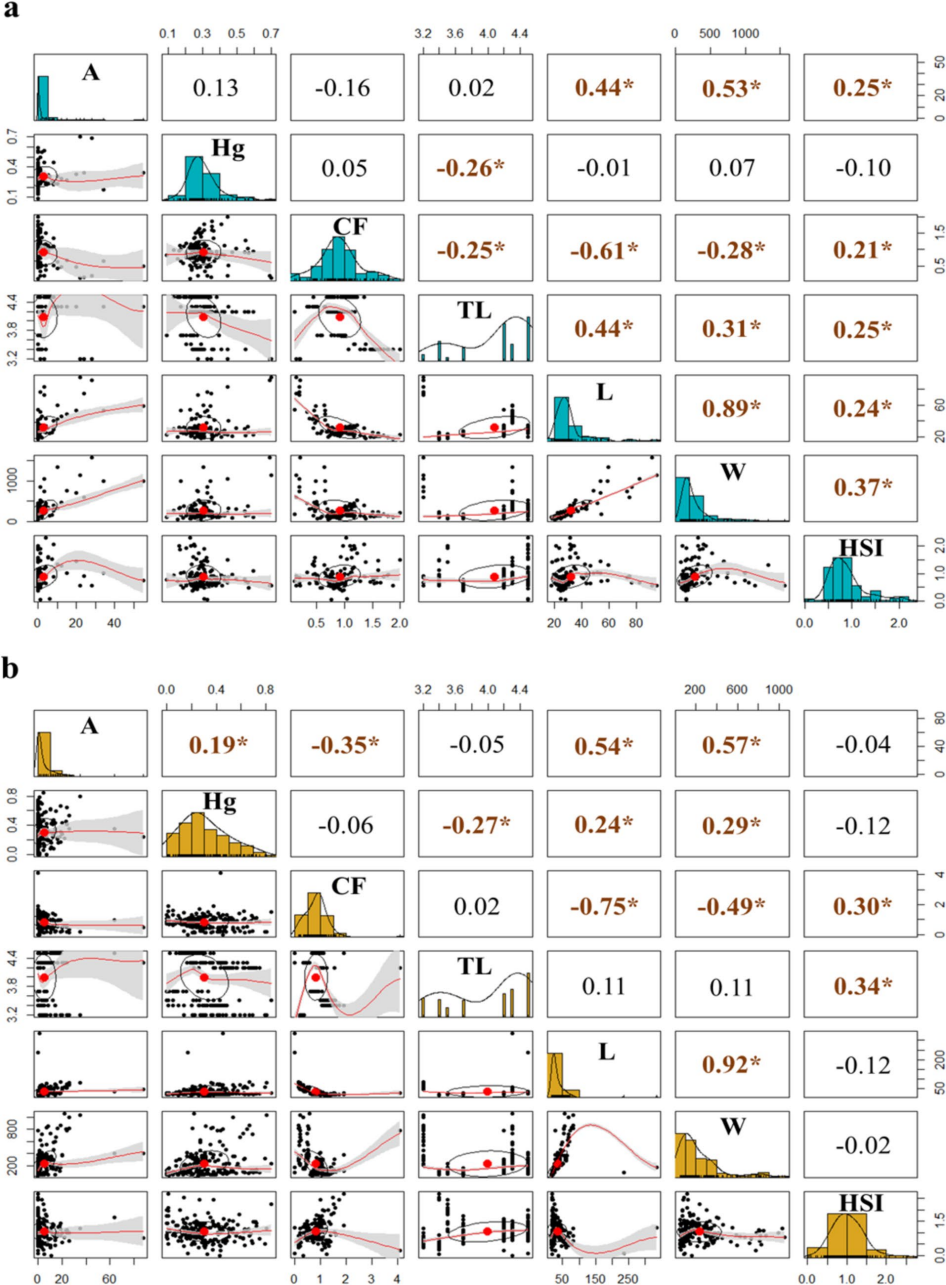
Spearman correlation (ρ) between Hg-T concentration, trophic level, morphometric and biometric parameters, and parasite index in fish during the rainy season (**a**) and dry season (**b**) in the San Jorge River, La Mojana Region, Sucre, Colombia. The correlation matrix plot shows bivariate scatter plots of the adjacent factors below the diagonal, histograms of the data distribution of the respective factors on the diagonal, and the Spearman correlation above the diagonal. Ellipses specify the direction of the correlation. Data with statistical significance levels are indicated with asterisks (*p* value < 0.05). Abbreviations: abundance (A), total mercury (Hg), condition factor (CF), trophic level (TL), total length (L), total weight (W), and hepatosomatic index (HSI)

A Spearman correlation analysis was conducted to explore preliminary associations between A, T-Hg, CF, TL, L, W, and HSI in both rainy and dry seasons, as shown in Fig. 6. Notably, this analysis used all data available without considering species as an additional variable.

During the rainy season, A exhibited significant positive correlations with L (ρ = 0.444; *p* < 0.05), W (ρ = 0.530; *p* < 0.05), and HSI (ρ = 0.251; *p* < 0.05). Mercury demonstrated a significant negative correlation with TL (ρ = -0.263; *p* < 0.05). Additionally, CF showed significant negative correlations with TL (ρ = -0.251; *p* < 0.05), L (ρ = -0.615; *p* < 0.05), and W (ρ = -0.281; *p* < 0.05), and a positive correlation with HSI (ρ = 0.211; *p* < 0.05). The highest correlation was found between L and W (ρ = 0.895; *p* < 0.05).

During the dry season, A showed positive correlation with T-Hg (ρ = 0.189; *p* < 0.05), L (ρ = 0.543; *p* < 0.05), and W (ρ = 0.572; *p* < 0.05), and a negative correlation with CF (ρ = -0.347; *p* < 0.05). Mercury exhibited positive correlations with L (ρ = 0.237; *p* < 0.05) and W (ρ = 0.291; p < 0.05), and a negative correlation with TL (ρ = -0.266; *p* < 0.05). Moreover, CF showed a positive correlation with HSI (ρ = 0.305; *p* < 0.05), and negative correlations with L (ρ = -0.745; *p* < 0.05) and W (ρ = -0.487; *p* < 0.05). The strongest correlation was again observed between L and W (ρ = 0.917; *p* < 0.05). When adjusting for TL, the partial Spearman correlation between A and T-Hg was ρ = 0.164 (*p* = 0.021), indicating these variables maintain a weak but statistically significant association independent of the TL.

### Principal Component Analysis (PCA)

To further explore the relationships among variables, a PCA was conducted to reduce dimensionality and summarize data variability. The variables from the correlation matrix were utilized, aimed at refining modeling precision. Additionally, cross-correlations among the principal components were scrutinized to juxtapose them against direct correlations. This procedure yielded seven principal components (PCs). The initial two PCs account for 50.7% of data variability, with 33.5% and 17.2%, for the first and second, respectively, and eigenvalues exceeding 1 (Fig. S4).

**Fig. 7:**
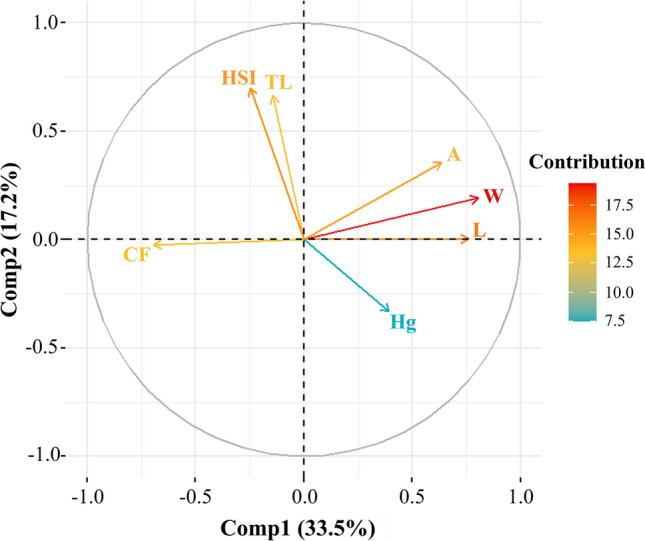
Principal Component analysis (PCA) biplot of Hg-T concentration, trophic level, morphometric and biometric parameters, and parasite index in fish from the San Jorge River in La Mojana Region, Sucre (Colombia). Abbreviations: trophic level (TL), abundance (A), total mercury (Hg), total weight (W), total length (L), hepatosomatic index (HSI), and condition factor (CF)

However, certain findings from the correlation matrix were validated through PCA (Fig. 7). The analysis identified an association among A, W, and L, suggesting that larger fish tend to exhibit higher parasite abundance. The correlation is stronger between L and W, as both increase simultaneously; heavier fish tend to be longer.

On the other hand, Hg correlates positively with A, indicating that a greater presence of Hg is associated with an increase in parasite abundance. Similarly, Hg shows an association with L and W, suggesting that larger and heavier fish have greater T-Hg concentrations. In contrast, CF exhibits an inverse association with Hg, A, L, and W, indicating that an increase in Hg concentration can deteriorate fish health, and this concentration tends to be higher in fish with greater W and L values.

Furthermore, HSI and TL show an association with each other, suggesting that fish with higher HSI values tend to occupy a higher TL. Trophic level showed a negative association with Hg, suggesting that carnivorous fish at higher TL may have lower Hg concentrations, potentially due to differing bioaccumulation patterns or detoxification mechanisms. These findings were similar to those observed in the correlation analysis, thereby reinforcing the results.

### Multiple Linear Regression (MLR) Analysis

A MLR analysis (*p* < 0.05) was conducted to assess the relationship between fish parasitic abundance and several predictor variables, including CF, T-Hg, and TL. The analysis revealed that CF and Hg significantly influenced parasitic abundance, with estimated coefficients of -5.385 (*p* < 0.001) and 6.109 (*p* = 0.037), respectively. These findings underscore the importance of considering multiple factors when analyzing fish populations and their health in aquatic environments. Furthermore, the Akaike Information Criterion (AIC) was used to identify the best-fit model among competing options in the multiple regression analysis (Table 2). Model selection was guided by the lowest AIC values obtained through stepwise regression.

**Table 2:** Adjusted R^2^ values and standard errors (SE) for factors in the best-fit models, as determined by Akaike Information Criterion values using the backward selection technique in multiple regression analyses

Models	Independent Variables	Estimate	Std. Error	*p* value	AIC	Adjusted R^2^	Overall *p* value
**1** A ~ T-Hg + CF + TL	Intercept	6.324	4.523	0.163	1404.01	0.072	0.006
	Hg	6.148	2.955	0.038*			
	CF	-5.378	1.139	< 0.001*			
	TL	0.095	1.012	0.925			
**2** A ~ T-Hg + CF	Intercept	6.728	1.443	< 0.001*	1402.64	0.075	< 0.001
	Hg	6.109	2.921	0.037*			
	CF	-5.385	1.134	< 0.001*			

The dependent variable in this analysis is A (parasitic abundance). *. Significance: *p* < 0.05

Abbreviations: Parasitic abundance (A), Total-Hg concentration (T-Hg), condition factor (CF), trophic level (TL), and Akaike Information Criterion (AIC)

The results indicate that the best-fit model (AIC = 1402.64) identified both Hg and CF as significant predictors of parasitic abundance, with the adjusted coefficients confirming the statistical significance of these effects (Table 2). Although TL was included in the initial model and showed significance in the unadjusted analysis, it was excluded from the final model during the backward elimination process, as its contribution was minimal and its removal improved the model's AIC score. The adjusted R^2^ value of 0.075 suggests that approximately 7.5% of the variability in parasitic abundance is explained by the combined effects of CF and Hg. While this model provides valuable insights, the relatively low R^2^ indicates that other unmeasured biological and environmental factors may also influence parasitic abundance, emphasizing the complexity of host–parasite interactions in aquatic ecosystems.

### Histological Analysis

**Fig. 8:**
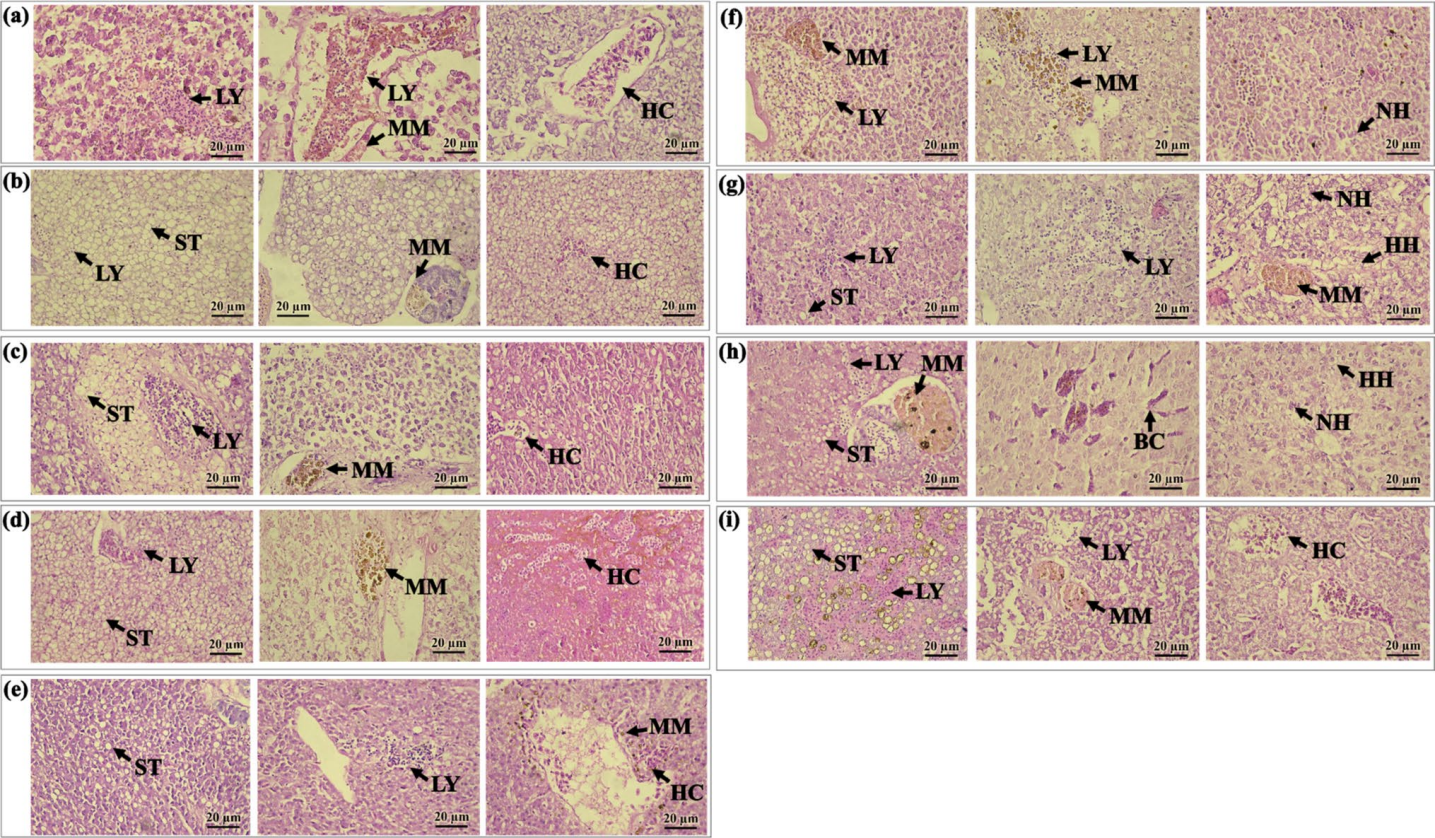
Liver histopathology in some fish species. HE 40X. (**a**) *H. malabaricus*, (**b**) *P. surinamensis*, (**c**) *P. magdaleniatum*, (**d**) *S. cuspicaudus*, (**e**) *C. magdalenae*, (**f**) *P. virginicus*, (**g**) *T. insignis*, (**h**) *C. kraussii*, (**i**) *S. macrurus*. Abbreviations: lipid vacuolization—steatosis (ST), Inflammatory lymphocytic infiltrates (LY), inflammation with melanomacrophages (MM), hepatic congestion (HC), nuclear hypertrophy (NH), hypertrophy of hepatocytes (HH), blood congestion (BC), hypertrophy of hepatocytes (HH)

Histological evaluation revealed the presence of ten categories of hepatic changes or alterations in all examined specimens (Fig. S5). These included lipid vacuolization (steatosis) (72%), lymphocytic inflammatory infiltrates (89%), inflammation with melanomacrophages (64%), nuclear hypertrophy (47%), blood congestion (39%), hepatic congestion (31%), hepatocyte hypertrophy (binucleated, multinucleated) (28%), apoptosis (14%), fibrosis of blood vessels with thickened fibrous wall (11%), and necrosis (6%) (Table S3). Some fish displayed an excessive accumulation of fat, where lipid-like vacuoles filled most of the cytoplasmic space in hepatocytes (Fig. 8). Additionally, the presence of parasitic cysts was observed in *P. magdaleniatum* (Fig. S5).

**Fig. 9:**
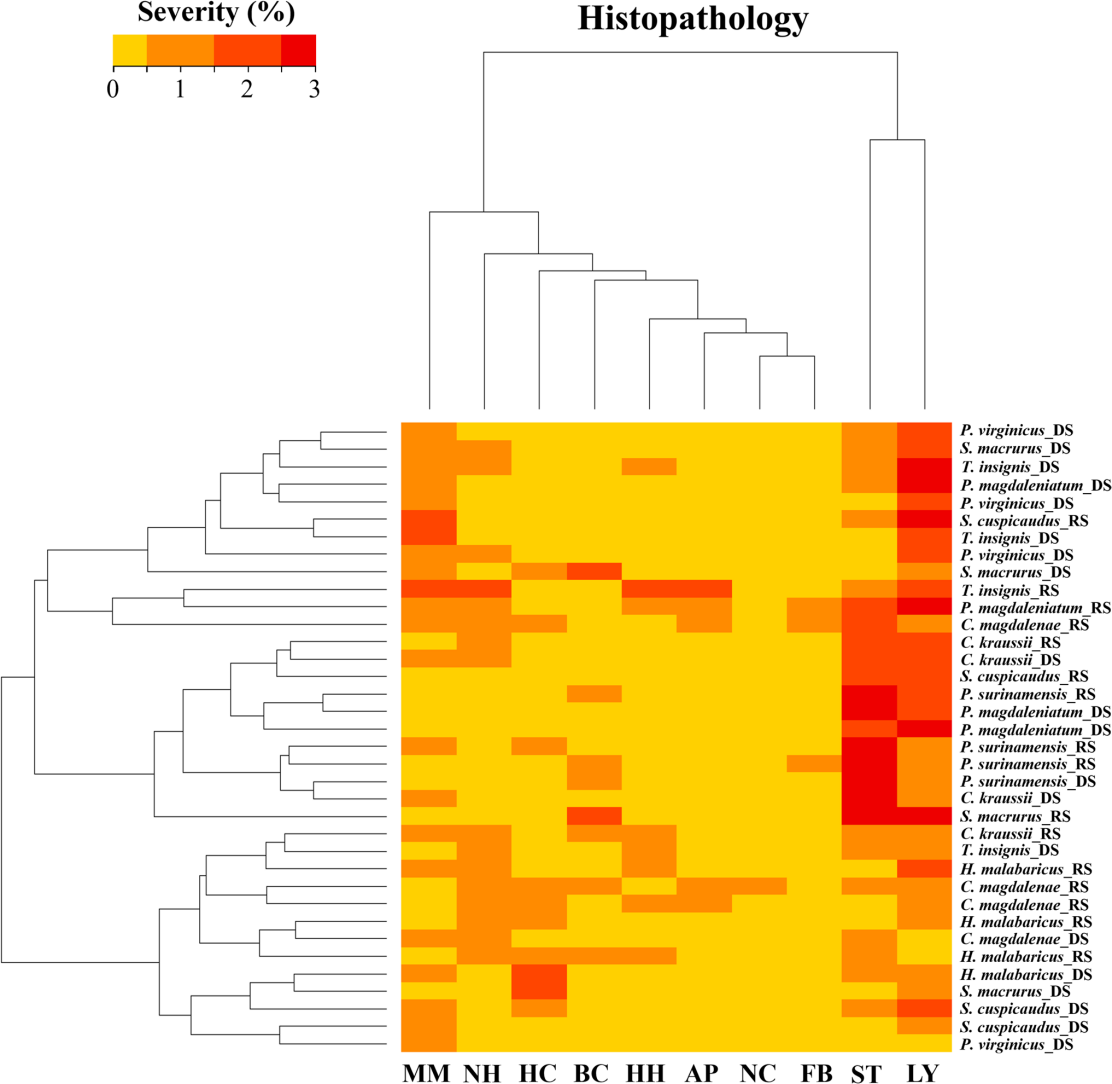
Heatmap viewer of histopathological alteration of liver tissue in fish species from San Jorge River in La Mojana Region, Sucre (Colombia). Abbreviations: Steatosis (ST), inflammatory lymphocytic infiltrates (LY), inflammation with melanomacrophages (MM), fibrosis of blood vessels with thickened fibrous wall (FB), hypertrophy of hepatocytes (HH), nuclear hypertrophy (binucleated. multinucleated) (NH), necrosis (NC), apoptosis (AP), hepatic congestion (HC), blood congestion (BC)

Liver histopathology for all species was clustered in a heatmap based on Euclidean distance to visualize the severities of liver alteration in fish. A visual representation of the relationships between 40 specimen and 10 liver histopathology categories, grouped into two distinct clusters, is provided in Fig. 9. Liver histopathologies such as steatosis were evidenced in *P. surinamensis*. *C. kraussii*, *P. magdaleniatum*, and *S. macrurus*. Likewise, severe inflammatory lymphocytic infiltrates were observed in *S cuspicaudus*, *P. magdaleniatum*, *S. macrurus*, and *T. insignis*. Other liver alterations such as inflammation with melanomacrophages, fibrosis of blood vessels with thickened fibrous wall, hepatocyte hypertrophy, nuclear hypertrophy, such as binucleated or multinucleated, necrosis, apoptosis, hepatic congestion, blood congestion, and parasite cyst, were observed in other specimens at various severity levels (Fig. S5).

### Risk-Based Fish Consumption Limits

Estimated Daily Intake, HQ, and CRmw values are presented for various fish species collected from the San Jorge River in San Marcos, located in the La Mojana region of Sucre, Colombia (Table S4). A CRmw value of 0 in the table indicates that the species should not be consumed even once a week due to potential risks. Analysis of the HQ values reveals that certain species pose higher risks for human consumption.

**Fig. 10:**
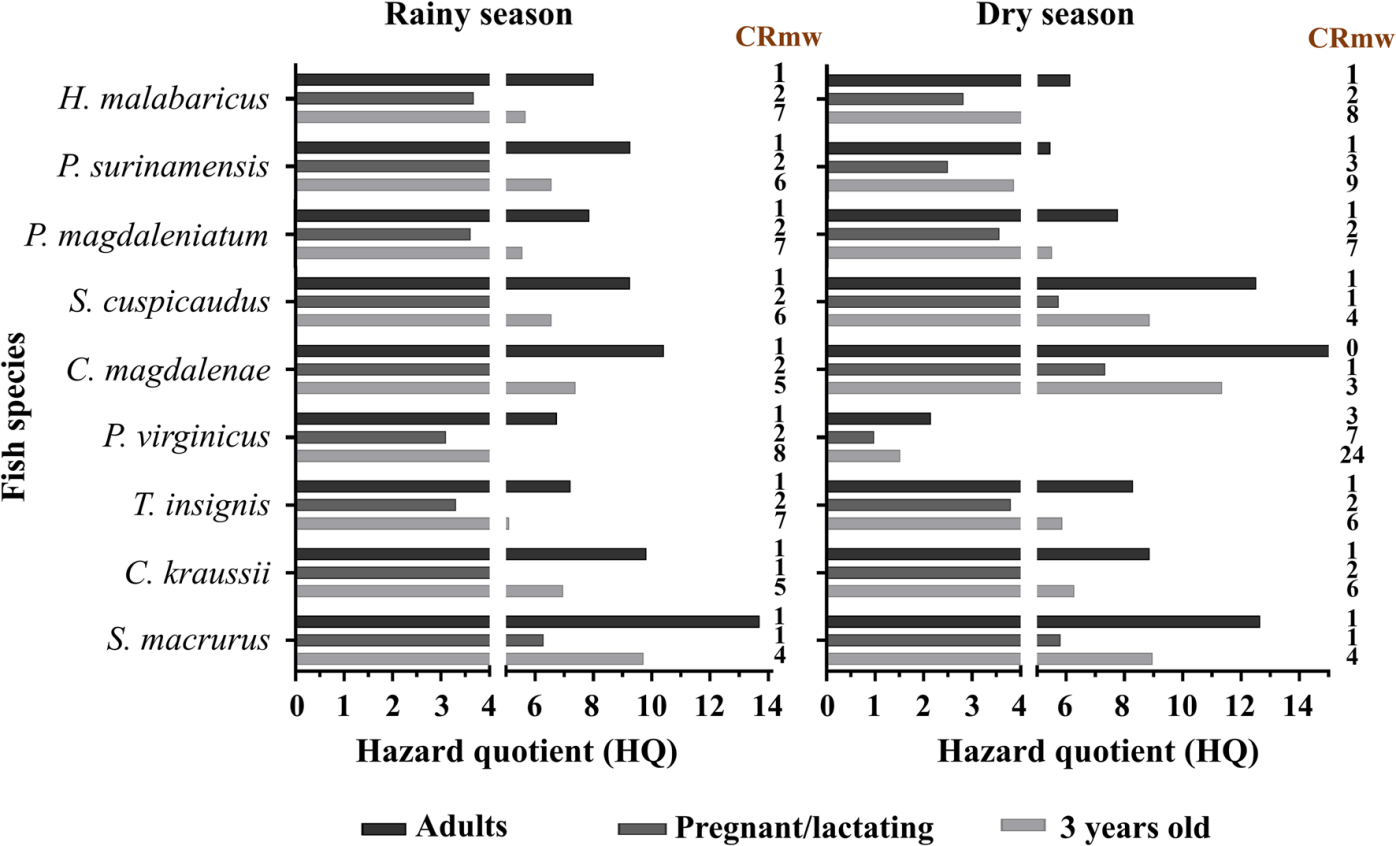
Hazard Quotient (HQ) and maximum allowable rates of fish consumption (CRmw) for adults, pregnant/lactating women, and children aged 3 years old, based on fish from the San Jorge River, La Mojana, Sucre (Colombia).

The calculated HQ values for the general population (Fig. 10, and Table S4) indicate that all fish species may pose a risk to human health, as HQ values greater than 1 were observed in the analyzed samples. This information, coupled with the CRmw results, suggests that in general, fish species suitable for consumption by the population of La Mojana include *P. virginicus*, which has the highest CRmw value during both rainy and dry seasons, indicating it can be consumed more frequently without significant health risks. Conversely, *C. magdalenae* has a CRmw value of 0 during the dry season, indicating that its consumption is not recommended during that time due to potential health risks.

## Discussion

In La Mojana, there are streams that connect to ‘muddy complexes’ (floodplain wetlands) which receive overflow from rivers during flood periods. Although no gold mining activities take place within the area itself, mercury-laden waste from nearby mining regions is carried downstream by streams and currents during the rainy season (Cruz-Esquivel et al. 2023). Therefore, some fish samples have been observed to exceed the maximum allowable limit of Hg established by the World Health Organization (0.5 mg/kg) (Romero-Suárez et al. 2022).

*C. kraussii* stands out for presenting the highest condition factor (1.57 ± 0.05) and *S. macrurus* the lowest (0.17 ± 0.01), coinciding with previous findings reported by Olivero-Verbel and Caballero-Gallardo (2013), where *C. kraussii* presented 1.63 ± 0.01 and *S. macrurus* 0.20 ± 0.02. Similarly, *T. insignis* exhibited the highest hepatosomatic index during both the rainy (1.79 ± 0.19%) and dry (1.73 ± 0.13%) seasons, aligning with previous reports by the same authors.

The overall presence of *Contracaecum* nematodes in this study aligns with previous findings in tropical freshwater ecosystems (Olivero-Verbel and Caballero-Gallardo 2013; Abd-ELrahman et al. 2023). While prevalence and abundance differed between the rainy and dry seasons, these seasonal variations may reflect changes in environmental conditions (such as water temperature, flow rate, or fish density) that can influence parasite transmission. Similar trends have been reported for other freshwater fish species, including *Oreochromis niloticus* (Nile tilapia) (Abd-ELrahman et al. 2023) suggesting that water availability and habitat structure can play a critical role in nematode proliferation.

The higher infection rates observed during the dry season similar to those reported by Uruku and Adikwu (2017) may be linked to the complex hydrological dynamics of La Mojana (Cruz-Esquivel et al. 2023). The reduced water flow during the dry season may promote closer contact between hosts and parasites, facilitating higher parasite transmission. In contrast, increased water volumes during the rainy season can disperse both hosts and parasites, decreasing their interactions and reducing infection prevalence.

Therefore, infection may be higher in the dry season than in the rainy season due to eutrophication factors, which can elevate parasitism by increasing parasite abundance. Eutrophication leads to algae blooming toward the end of the rainy season, resulting in a variety of both species and parasites, thereby increasing fish parasite infection and ultimately leading to parasite maturation during the dry season (Uruku and Adikwu 2017). In La Mojana, studies addressing biological and chemical contamination in the region have also been reported. These reports have revealed the high prevalence of parasites, such as *Contracaecum* sp., in fish and birds, linking it to organic matter and eutrophication in the region (Consuegra-Solórzano 2009; Wadnípar-Cano 2013). Another factor contributing to higher parasitic prevalence during the dry season is the absence of precipitation and the significantly reduced water flow and volume, leading to much greater contact between parasites and fish, resulting in a relatively higher prevalence. This finding aligns with the observations of Khalid et al. (2021) and Uruku and Adikwu (2017) and may help explain the results observed in this study. On the other hand, Iyaji and Yaro (2016) report that in the tropics, especially in the confluence area of the Niger-Benue rivers, where temperatures remain high throughout the year with minimal or no fluctuations, the abundance of parasitic nematodes found throughout their study could be explained.

In Colombia, L3 larvae of *Contracaecum* sp. have been found in high parasitic prevalence of up to 100% in *Mugil incilis* (Lisa) from Cartagena Bay and Ciénaga El Totumo, representing a potential risk of Anisakidosis in communities where this fish is a preferred food source. The genus *Contracaecum* includes approximately 50 species with a worldwide distribution, which occurs naturally, likely through transportation in migratory birds and low specificity in choosing intermediate and definitive hosts (Anderson 2000; D'Amelio et al. 2007).

The length of *Contracaecum* nematodes in this study averaged 16.2 mm, similar to that reported by Choc-M et al. (2020) which averaged 15.7 mm, with a range of 1–18 mm. Morphological identification revealed characteristics of the genus *Contracaecum* larvae (Nematoda, Anisakidae). These analyses indicated that the nematodes in this study exhibited the typical and specific characteristics of L3 larvae, Tipo II, of the genus *Contracaecum*. These features are the presence of an intestinal caecum extending from the ventricle to the vicinity of the nerve ring and lips with a cuticular tooth between them as reported by Olivero-Verbel et al. (2011) y Vuić et al. (2022).

Parasitic nematodes of the genus *Contracaecum* (family Anisakidae) were identified in the fish examined during this study. Members of the Anisakidae family (*Anisakis*, *Pseudoterranova*, and *Contracaecum*) are widely distributed in marine and estuarine fish populations worldwide and have garnered attention because of their pathogenicity in humans (Mehrdana and Buchmann 2017). Their infection process is linked to the secretion of excretory-secretory (ES) proteins by the esophageal glands of the nematodes, which suppress or modulate the host immune system, facilitating parasite survival (Gahoi et al. 2019). In humans, the principal risk arises from ingesting raw or undercooked fish containing these larvae, potentially leading to anisakidosis or allergic responses. Although proper cooking or freezing neutralizes the infectivity of the larvae, certain ES proteins remain resistant to heat and other processing methods (Carballeda-Sangiao et al. 2014; Mehrdana and Buchmann 2017). Consequently, even well-cooked or canned fish may elicit an immune response in individuals who are sensitized to these allergens. Moreover, parasitic nematodes can adversely affect the overall health and nutritional value of infected fish, given their capacity to compromise physiological functions in the host (Joshi and Mishra 2022). Therefore, there is a need to adopt proper fish-handling and preparation practices (e.g., thorough cooking or freezing) to minimize infection risks. This approach recognizes the ecological complexity of host–parasite interactions in aquatic systems, where parasites can intersect with other environmental stressors to affect both animal and human health. Furthermore, it emphasizes the exploration of novel methods aimed at disrupting host–parasite relationships, particularly by suppressing parasite ES products, in order to identify new therapeutic targets for parasite control.

Building upon these insights into host–parasite interactions, the final hosts of *Contracaecum* species are piscivorous birds (mainly Pelecaniformes) associated with freshwater and marine environments, and seals worldwide, with *C. rudolphii*'s main final hosts being birds (Amato et al. 2006), as well as *C. microcephalum* (Bennett et al. 2022). However, *C. rudolphii* C has been found in freshwater fish (Mattiucci et al. 2020; Aqeele et al. 2024), and likewise, freshwater fish have been infected with larvae of *C. microcephalum* species (Mhaisen and Abdul-Ameer 2021). This nematode species has also been found in round goby, *Neogobius melanostomus*, from the Black Sea, revealing a high level of infection by anisakid juveniles, identified as *C. microcephalum* (Pronkina and Spiridonov 2020). Similar to the findings of this study, molecular identification confirms that the genus of the nematodes is *Contracaecum*, with high percentages of similarities observed with two species, *C. microcephalum* and *C. rudolphii* C.

The statistical analysis using Fisher's exact test revealed a significant association in the prevalence of parasitosis between the rainy and dry seasons among the studied fish species, including *P. magdaleniatum*, *H. malabaricus*, *P. surinamensis*, and *T. insignis*. These findings underscore the significant influence of seasonal environmental conditions on the dynamics of parasitic diseases in aquatic ecosystems. The observed variations suggest a sensitivity of the species to seasonal climatic conditions and their susceptibility to parasitic infection under different environmental circumstances. These associations may be related to changes in water temperature, the availability of food resources, and other environmental factors. These results highlight the importance of considering seasonal effects when evaluating the dynamics of parasitic diseases in aquatic ecosystems and emphasize the need for additional research to better understand these underlying mechanisms, consistent with the findings reported by Uruku and Adikwu (2017) and Iyaji and Yaro (2016).

The correlation analysis showed that larger fish tend to have a higher parasitic load. Similarly, fish with higher Hg levels are associated with greater parasite abundance, longer length, and greater weight. These higher Hg levels, often found in larger fish, can negatively impact fish health. Additionally, fish with higher HSI values tend to occupy a higher TL, indicating that fish at higher TL may have larger livers due to their diet and metabolic needs. Top predators tend to consume larger, energy-rich prey, which can lead to greater liver development (Olivero-Verbel and Caballero-Gallardo 2013).

While it is generally expected that Hg concentration is directly proportional to trophic level, with a positive correlation (Alcala-Orozco et al. 2020), the present study found the opposite, which is consistent with the findings of da Silva et al. (2020). Trophic level showed a negative association with Hg concentrations, suggesting that fish at higher TL may have lower Hg concentrations. This unexpected finding can be explained by several factors supported by previous studies.

According to de Castro Moraes et al. (2023), variations in Hg bioaccumulation among fish species can be influenced by factors such as diet composition, habitat differences, and regional Hg dynamics. For instance, *Triportheus elongatus*, an omnivorous species, exhibits fluctuating Hg concentrations due to its diverse diet and habitat range. Similarly, in the Mediterranean, regional differences in MeHg availability and local methylation processes have been shown to affect bioaccumulation in zooplankton, ultimately influencing Hg concentrations in higher TL fish (Buckman et al. 2018). However, Marrugo-Negrete et al. (2018a, b) reported that despite seasonal changes, fish feeding patterns and trophic positions remain relatively stable throughout the year, suggesting that nutrient availability and marsh dynamics might explain lower Hg concentrations in certain predatory species. Additionally, Zhang et al. (2022) observed that reduced Hg concentrations in fish populations from China were linked to overfishing and aquaculture practices, leading to lower TL and consequently reduced Hg accumulation. These findings suggest that factors beyond TL, such as regional Hg sources, methylation patterns, and species-specific detoxification mechanisms, play a critical role in Hg bioaccumulation patterns.

Colombia, one of the most biodiverse countries in the world, has been the focus of multiple studies evaluating Hg levels in fish across different regions. For instance, in the Cauca Department, 17 fish species were collected from various sites, with TL ranging from 2.0 to 4.0. Reported Hg concentrations varied by location: 0.17–2.53 µg/g in La Balsa (Cauca River), 0.25–0.68 µg/g in the Quinamayo River, and 0.01–0.14 µg/g in the Mazamorrero River and the San Miguel stream (Caballero-Gallardo et al. 2022). In Tadó (Department of Chocó, western Colombia), a total of 117 fish samples belonging to eight species (TL 2.0–4.5) were analyzed. The overall mean concentration of T-Hg in fish muscle was 0.25 (± 0.25) µg/g wet weight, with the highest average (0.46 ± 0.20 µg/g) found in *Hoplias malabaricus* and the lowest (0.10 ± 0.05 µg/g) in *Cynopotamus magdalenae* (Gutiérrez-Mosquera et al. 2021). Another study carried out in six municipalities in the Department of Bolívar (covering areas of La Mojana and Middle Magdalena) analyzed 62 fish. The T-Hg concentrations in muscle ranged from 0.03 to 0.37 µg/g. The highest average value (0.37 ± 0.10 µg/g) was recorded in *C. kraussii*, and the lowest (0.03 ± 0.01 µg/g) in *Prochilodus magdalenae*. For Middle Magdalena, the decreasing order of T-Hg was *S. cuspicaudus* > *P. magdaleniatum* > *P. magdalenae*, while in the Mojana region, the order was *C. kraussii* > *P. surinamensis* > *T. insignis* > *M. muyscorum* > *P. magdalenae* (Carranza-Lopez et al. 2019).

Compared with other regions, Hg contamination in fish from La Mojana is not an isolated phenomenon. Multiple studies have highlighted significant concerns regarding Hg in this area. Romero-Suarez et al. (2022) investigated Hg levels in fish muscle in San Marcos, a high-risk area due to intense human activity and pollution. Their findings revealed notable Hg concentrations in fish species, raising concerns about water quality and potential health risks for both humans and wildlife. Similarly, Guzmán Ospino (2021) reported elevated Hg concentrations in fish muscle from several localities in La Mojana, including Caimito, Guaranda, San Benito, and San Marcos-areas in close proximity to industrial and agricultural pollution sources. Moreover, the high concern in La Mojana extends beyond fish, as Hg presence has also been documented in other animal species, such as turtles (Meza-Martínez et al. 2020), bats (Calao-Ramos et al. 2021), and ducks (Buelvas-Soto et al. 2022), highlighting the metal’s movement throughout the trophic chain.

Collectively, these studies underscore a widespread issue of metal contamination in the region and reveal mounting concerns about Hg levels in La Mojana. Although research efforts have grown, systematic studies on Hg in fish across Colombia remain limited, hindering more extensive comparisons and a comprehensive understanding of contamination patterns nationwide.

In the context of La Mojana, Sucre, Colombia, these findings are particularly relevant. The region's rich biodiversity and varying water conditions create diverse habitats and dietary patterns among fish species. The interplay between different sources of Hg, including anthropogenic activities such as mining and natural processes, along with regional differences in nutrient availability, may contribute to the observed Hg concentrations in fish. Therefore, the lower Hg concentrations in higher TL fish in La Mojana could be attributed to similar bioaccumulation and detoxification mechanisms observed in other studies, highlighting the complex interactions between environmental factors and Hg dynamics in aquatic ecosystems.

On the other hand, the fish species commonly consumed in the region reveal concerning levels of Hg contamination during the rainy and dry seasons. Mercury is an environmental contaminant that causes acute and chronic damage to multiple organs. In fish, virtually all organic Hg is found in the form of MeHg, which has been associated with health issues in both animals and humans. Consequently, contaminants such as Hg and parasites can lead to alterations in the livers of fish, as reported by Guardiola et al. (2016), which is similar to what was found in this study. Some specimens showed excessive fat accumulation, where lipid-like vacuoles filled most of the cytoplasmic space of the hepatocytes, as observed in *P. surinamensis*. Mercury exposure and parasitism have been associated with liver alterations in fish, as reported by Dang et al. (2017) and De Troyer et al. (2016). These studies suggest that Hg exposure can cause substantial hepatic injury, which may be exacerbated by parasitic infections, indicating a complex interaction between environmental contaminants and parasitic health effects in fish. Similarly, Behrens et al. (2023) described severe liver inflammation in Baltic cod infected with *Contracaecum osculatum*, where high parasite loads were linked to granulomatous changes, cellular debris, vascular damage, and hemorrhages. The tissue damage observed appeared to be a defense response against nematode migration but resulted in hepatocyte destruction, potentially compromising liver functionality and overall fish health. The selection of T-Hg, CF, and TL as predictors in the statistical models was based on previous ecological and toxicological studies linking these variables to both metal bioaccumulation and parasitic dynamics in fish populations (Olivero-Verbel and Caballero-Gallardo 2013; Marrugo Negrete et al. 2018). These findings highlight the need for further studies to better understand the combined effects of pollutants and parasitic infections on fish health.

The risk assessment of Hg exposure based on fish consumption indicates that all examined species may pose a potential health risk (HQ > 1). Therefore, caution is advised regarding frequent consumption of these species, as excessive intake could increase health risks associated with Hg exposure. Interestingly, the associations observed between Hg levels in fish muscle tissue, nematode infestation, and TL level suggest that reducing fish consumption due to Hg exposure could also potentially lower the risk of parasite exposure. Further research is needed to clarify this relationship and better understand the interactions between chemical contaminants and parasitic infections within the aquatic ecosystem of La Mojana, Colombia. These results are consistent with those reported by Marrugo-Negrete et al. (2020).

## Conclusion

Fish from the Mojana region of Sucre, originating from the San Jorge River, present a high prevalence and abundance of nematode parasites, primarily *Contracaecum microcephalum* voucher and *Contracaecum rudolphii* C. Additionally, these fish contain Hg, a contaminant known for its potential to cause hepatic alterations, similar to the effects observed with parasitic infections. Interestingly, lower T-Hg concentrations were found in higher trophic level fish, which may be explained by bioaccumulation and detoxification mechanisms specific to the local ecosystem. These findings underscore the complex interactions between environmental stressors, such as T-Hg and parasitism, and their combined impact on aquatic organisms.

The risk assessment conducted indicates that all analyzed fish species may pose a potential health risk due to Hg exposure (HQ > 1). Therefore, caution is advised regarding frequent consumption of these species, as excessive intake could increase health risks associated with mercury exposure. However, this recommendation should be viewed with caution, as it is based on preliminary data and does not account for other contaminants or parasitic risks. Further research is essential to comprehensively evaluate the combined effects of Hg, parasitism, and additional environmental factors on fish health and human safety.

## Supplementary Information

Below is the link to the electronic supplementary material.

Supplementary file1 (DOCX 8829 KB)

## Data Availability

Data will be made available on request.
