# Supplementary material for: Presence of Nematodes, Mercury Concentrations, and Liver Pathology in Carnivorous Freshwater Fish from La Mojana, Sucre, Colombia: Assessing Fish Health and Potential Human Health Risks
Source: Arch Environ Contam Toxicol. 2025 Feb 20;88(2):189–209. doi: 10.1007/s00244-025-01117-w (PMC11870952; doi:10.1007/s00244-025-01117-w)
Supplement: Supplementary file 1 — Supplementary file1 (DOCX 8829 KB) [file 244_2025_1117_MOESM1_ESM.docx]

**Presence of nematodes, Mercury Concentrations, and Liver Pathology in Freshwater Fish from La Mojana, Sucre, Colombia: Assessing Fish Health and Potential Human Health Risks**

Fuentes-Lopez Katerin^1,2^, Caballero-Gallardo Karina^1,2^, Olivero-Verbel Jesus^1^*

1. Environmental and Computational Chemistry Group, School of Pharmaceutical Sciences, Zaragocilla Campus, University of Cartagena, Cartagena 130014, Colombia

2. Functional Toxicology Group, School of Pharmaceutical Sciences, Zaragocilla Campus, University of Cartagena, Cartagena 130014, Colombia

*. Corresponding Author:

Prof. Jesus Olivero-Verbel, Ph.D.

Environmental and Computational Chemistry Group

School of Pharmaceutical Sciences

University of Cartagena

Cartagena, Colombia

Tel: 318-535-9815

Fax: 57-(5)-6699771

E-mail: [joliverov@unicartagena.edu.co](mailto:joliverov@unicartagena.edu.co)

**Contenido**

1. **Supplementary Table S1**. Sequence of primers used in nematode identification.
2. **Supplementary Table S2**. T-Hg concentration in fish species from La Mojana Region, Sucre.
3. **Supplementary Table S3**. Histopathological alteration of liver tissue in fish species from San Jorge River in La Mojana Region. Sucre (Colombia).
4. **Supplementary Figure S1**. Walter-Lieth climatic diagram based on historical climate data for San Marcos, La Mojana-Sucre (Colombia).
5. **Supplementary Figure S2**. Morphological characteristics of the nematode found in fish species from La Mojana, Colombia. (A) *P. surinamensis*, (B) *S. cuspicaudus*, (C) *C. magdalenae*, (D) *C. kraussii*, (E) *S. macrurus*.
6. **Supplementary Figure S3**. T-Hg concentration in fish species between season from La Mojana Region, Sucre.
7. **Supplementary Figure S4**. Principal Components scree plot showing the percentage of explained variances.
8. **Supplementary Figure S5**. Histopathology in fish species liver HE40X. Abbreviations: Inflammatory lymphocytic infiltrates (LY), necrosis (NC), hepatic congestion (HC), and parasite cyst (PC) in P. magdaleniatum. Inflammation with melanomacrophages (MM), Nuclear hypertrophy (NH), and blood congestion (BC) in C. kraussii. Lipid vacuolization – steatosis (ST) in P. surinamensis. Hypertrophy of hepatocytes (HH) in H. malabaricus. Apoptosis (AP), and fibrosis of blood vessels with thickened fibrous wall (FB) in C. magdalenae.
9. **Supplementary Table S4**. Hazard Quotient (HQ) and maximum allowable rates of fish consumption (CRmw) for adults, pregnant/lactating women, and children aged 3, 4, and 5 years, based on fish from the San Jorge River in San Marcos, La Mojana, Sucre (Colombia).

**Table S1.** Sequence of primers for nematode primers

| **Gene name** | **Forward (5’ 🡪 3’)** | **Reverse (5´🡪 3’)** | **Amplicon size (bp)** | **Reference** |
| --- | --- | --- | --- | --- |
| RrnS | TTGTTCCAGAATAATCGGCTAGACTT | TCTACTTTACTACAACTTACTCC | 485 | (D'Amelio et al. 2007) |

**Table S2**. T-Hg concentration in fish species from La Mojana Region, Sucre.

| Specie | Rainy season | Dry season | t-Student (t), p-value |
| --- | --- | --- | --- |
| *Hoplias malabaricus* | 0.249±0.01 | 0.207±0.02 | 1.609 *p*=0.117 |
| *Plagioscion surinamensis* | 0.271±0.01 | 0.184±0.01 | **5.559 *p*<0.001** |
| *Pseudoplatystoma magdaleniatum* | 0.266±0.03 | 0.263±0.02 | 0.100 *p*=0.921 |
| *Sorubim cuspicaudus* | 0.313±0.02 | 0.423±0.03 | **2.739 *p*=0.011** |
| *Cynopotamus magdalenae* | 0.352±0.02 | 0.542±0.03 | **6.206 *p*<0.001** |
| *Polydactylus virginicus* | 0.228±0.02 | 0.073±0.02 | **4.693 *p*<0.001** |
| *Trachelyopterus insignis* | 0.244±0.03 | 0.280±0.07 | 0.238 *p*=0.816 |
| *Caquetaia kraussii* | 0.332±0.02 | 0.299±0.03 | 0.920 *p*=0.363 |
| *Sternopygus macrurus* | 0.464±0.08 | 0.428±0.04 | 0.411 *p*=0.684 |

Significance: *p*<0.05

**Table S3**. Histopathological alteration of liver tissue in fish species from San Jorge River in La Mojana Region. Sucre (Colombia).

| **Specie** | **ST** | **LY** | **MM** | **FB** | **HH** | **NH** | **NC** | **AP** | **HC** | **BC** |
| --- | --- | --- | --- | --- | --- | --- | --- | --- | --- | --- |
| *H. malabaricus* | 1 | 0 | 0 | 0 | 1 | 1 | 0 | 0 | 1 | 1 |
| *H. malabaricus* | 0 | 2 | 1 | 0 | 1 | 1 | 0 | 0 | 0 | 0 |
| *H. malabaricus* | 1 | 1 | 0 | 0 | 0 | 1 | 0 | 0 | 1 | 0 |
| *H. malabaricus* | 1 | 1 | 1 | 0 | 0 | 0 | 0 | 0 | 2 | 0 |
| *P. surinamensis* | 3 | 2 | 0 | 0 | 0 | 0 | 0 | 0 | 0 | 1 |
| *P. surinamensis* | 3 | 1 | 1 | 0 | 1 | 1 | 0 | 0 | 1 | 0 |
| *P. surinamensis* | 3 | 1 | 0 | 1 | 0 | 0 | 0 | 0 | 1 | 1 |
| *P. surinamensis* | 3 | 1 | 0 | 0 | 0 | 0 | 0 | 0 | 0 | 1 |
| *P. magdaleniatum* | 2 | 3 | 1 | 1 | 1 | 1 | 0 | 1 | 1 | 1 |
| *P. magdaleniatum* | 3 | 2 | 0 | 0 | 0 | 0 | 0 | 0 | 0 | 0 |
| *P. magdaleniatum* | 2 | 3 | 0 | 0 | 0 | 0 | 0 | 0 | 0 | 0 |
| *P. magdaleniatum* | 1 | 3 | 1 | 0 | 0 | 0 | 0 | 0 | 0 | 0 |
| *S.cuspicaudus* | 1 | 3 | 2 | 0 | 0 | 0 | 0 | 0 | 0 | 0 |
| *S.cuspicaudus* | 2 | 2 | 0 | 0 | 0 | 0 | 0 | 0 | 0 | 0 |
| *S.cuspicaudus* | 0 | 1 | 1 | 0 | 0 | 0 | 0 | 0 | 0 | 0 |
| *S.cuspicaudus* | 1 | 2 | 1 | 0 | 0 | 0 | 0 | 0 | 1 | 0 |
| *C. magdalenae* | 2 | 1 | 1 | 1 | 0 | 1 | 0 | 1 | 2 | 0 |
| *C. magdalenae* | 1 | 1 | 0 | 0 | 0 | 1 | 1 | 1 | 1 | 1 |
| *C. magdalenae* | 0 | 1 | 0 | 0 | 1 | 1 | 0 | 1 | 1 | 1 |
| *C. magdalenae* | 1 | 0 | 1 | 0 | 0 | 1 | 0 | 0 | 1 | 0 |
| *P. virginicus* | 0 | 2 | 2 | 0 | 0 | 2 | 0 | 0 | 0 | 0 |
| *P. virginicus* | 0 | 0 | 1 | 0 | 0 | 0 | 0 | 0 | 0 | 0 |
| *P. virginicus* | 0 | 2 | 2 | 0 | 0 | 0 | 0 | 0 | 0 | 0 |
| *P. virginicus* | 1 | 2 | 1 | 0 | 1 | 1 | 0 | 0 | 0 | 0 |
| *T. insignis* | 1 | 2 | 2 | 0 | 2 | 2 | 0 | 2 | 0 | 0 |
| *T. insignis* | 1 | 1 | 0 | 0 | 1 | 1 | 0 | 0 | 1 | 0 |
| *T. insignis* | 0 | 2 | 2 | 0 | 0 | 0 | 0 | 0 | 0 | 0 |
| *T. insignis* | 1 | 3 | 1 | 0 | 1 | 1 | 0 | 0 | 0 | 0 |
| *C. kraussii* | 1 | 1 | 1 | 0 | 1 | 1 | 0 | 0 | 0 | 1 |
| *C. kraussii* | 2 | 2 | 0 | 0 | 0 | 1 | 1 | 0 | 0 | 1 |
| *C. kraussii* | 2 | 2 | 1 | 0 | 0 | 1 | 0 | 0 | 0 | 0 |
| *C. kraussii* | 3 | 1 | 1 | 0 | 0 | 0 | 0 | 0 | 0 | 0 |
| *S. macrurus* | 3 | 3 | 0 | 0 | 0 | 0 | 0 | 0 | 0 | 2 |
| *S. macrurus* | 0 | 1 | 1 | 0 | 0 | 0 | 0 | 0 | 2 | 0 |
| *S. macrurus* | 0 | 2 | 1 | 0 | 0 | 0 | 0 | 0 | 1 | 2 |
| *S. macrurus* | 1 | 2 | 1 | 0 | 0 | 1 | 0 | 0 | 0 | 0 |

Abbreviations: ST, Lipid vacuolization (steatosis). LY, inflammatory lymphocytic infiltrates. MM, inflammation with melanomacrophages. FB, fibrosis of blood vessels with thickened fibrous wall. HH, hypertrophy of hepatocytes. NH, nuclear hypertrophy (binucleated, multinucleated). NC, necrosis. AP, apoptosis. HC, hepatic congestion. BC, blood congestion.


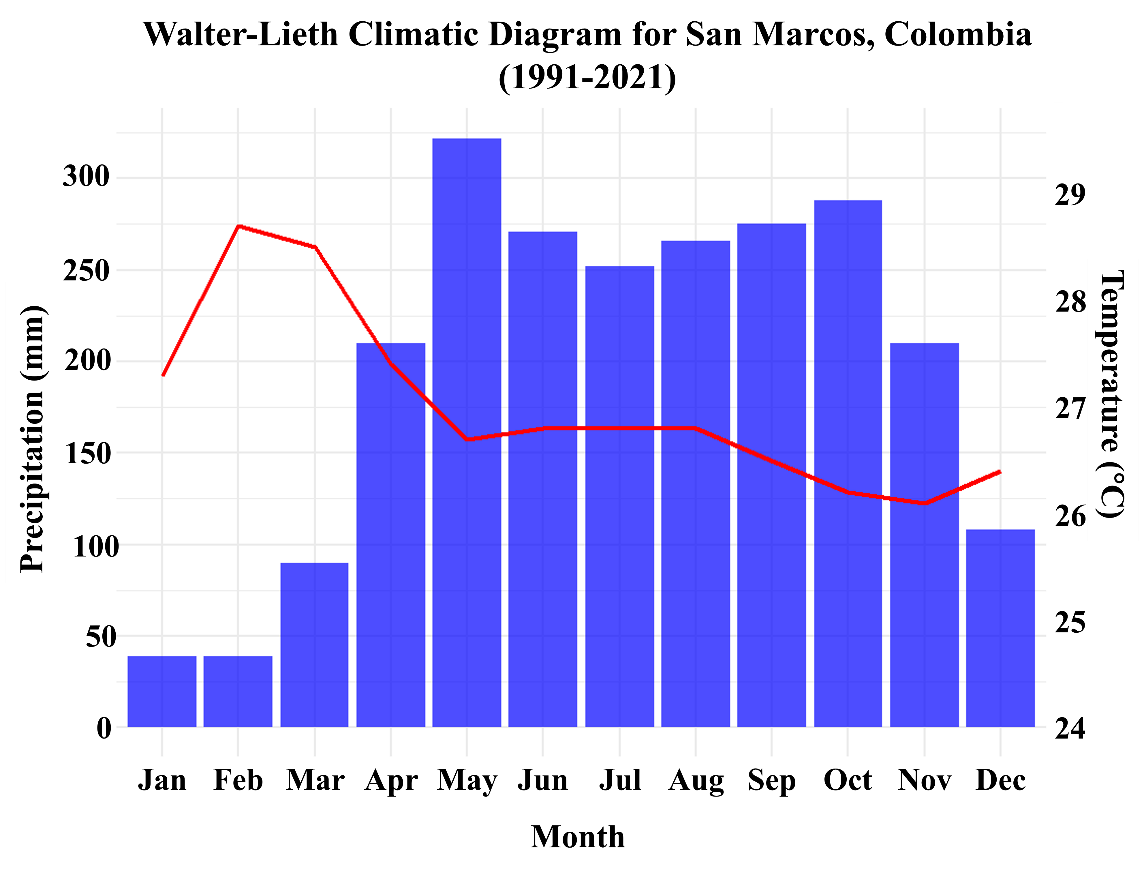


**Figure S1**. Walter-Lieth climatic diagram based on historical climate data for San Marcos, La Mojana-Sucre (Colombia).

**
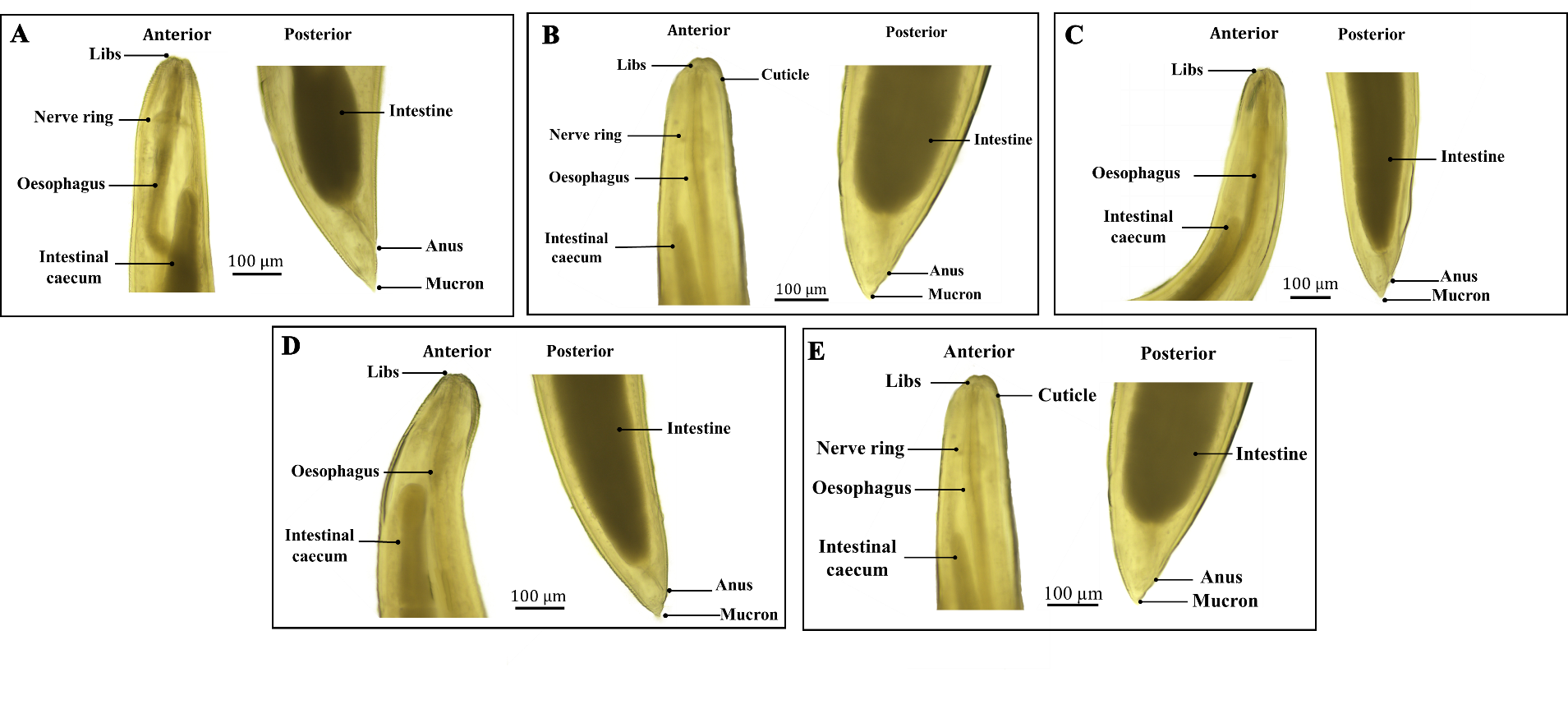
**

**Figure S2.** Morphological characteristics of the nematode found in fish species from La Mojana, Colombia. (A) *P. surinamensis*, (B) *S. cuspicaudus*, (C) *C. magdalenae*, (D) *C. kraussii*, (E) *S. macrurus*.

**
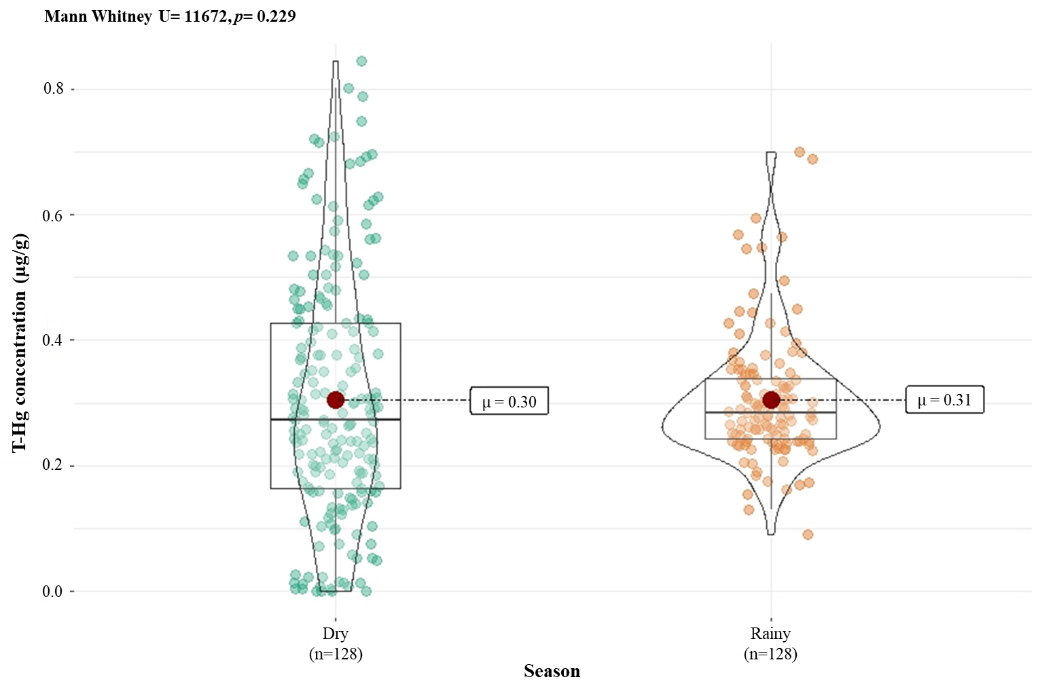
**

**Figure S3**. T-Hg concentration in fish species between season from La Mojana Region, Sucre.


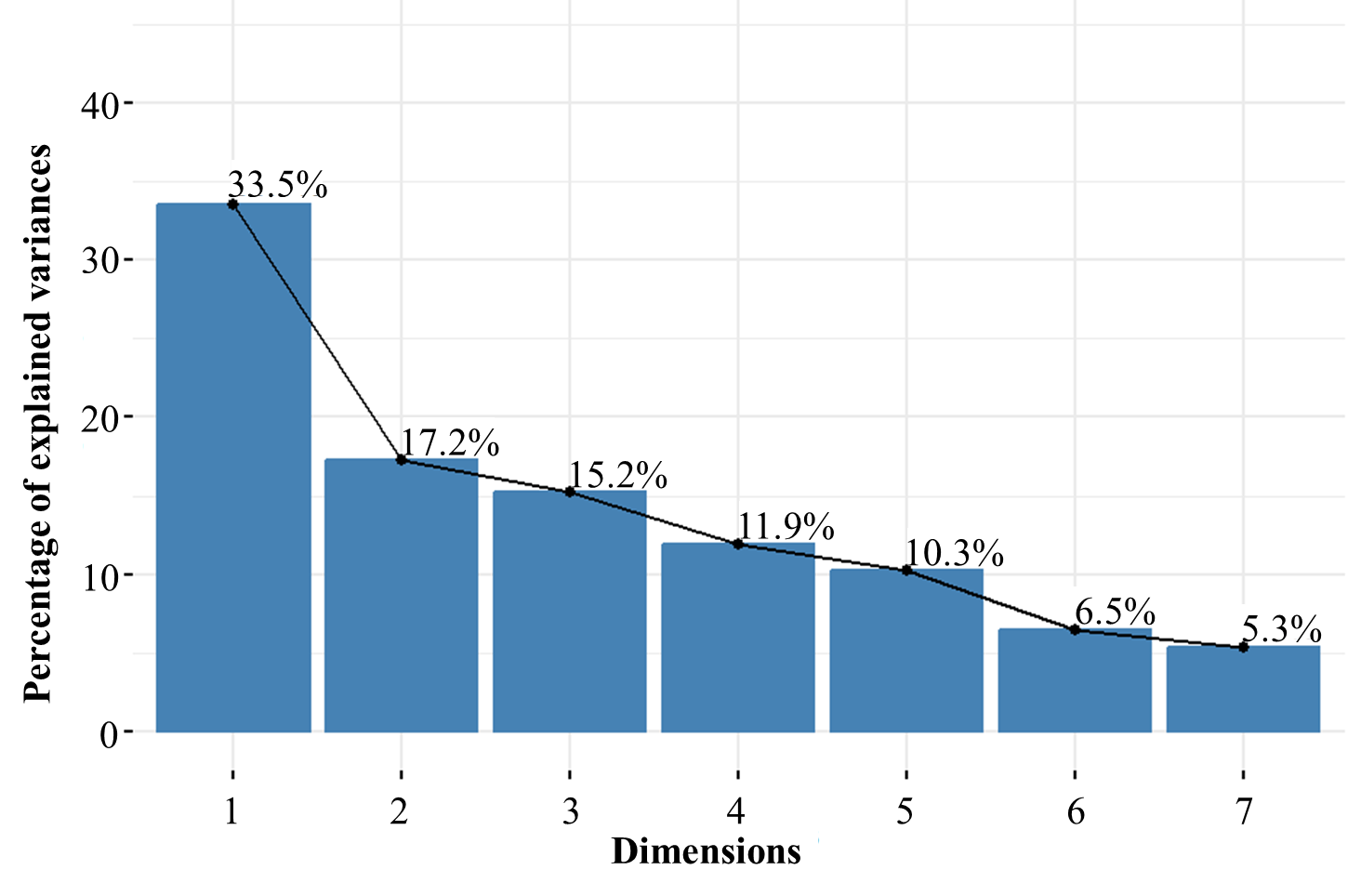


**Figure S4.** Principal Components scree plot showing the percentage of explained variances.

**
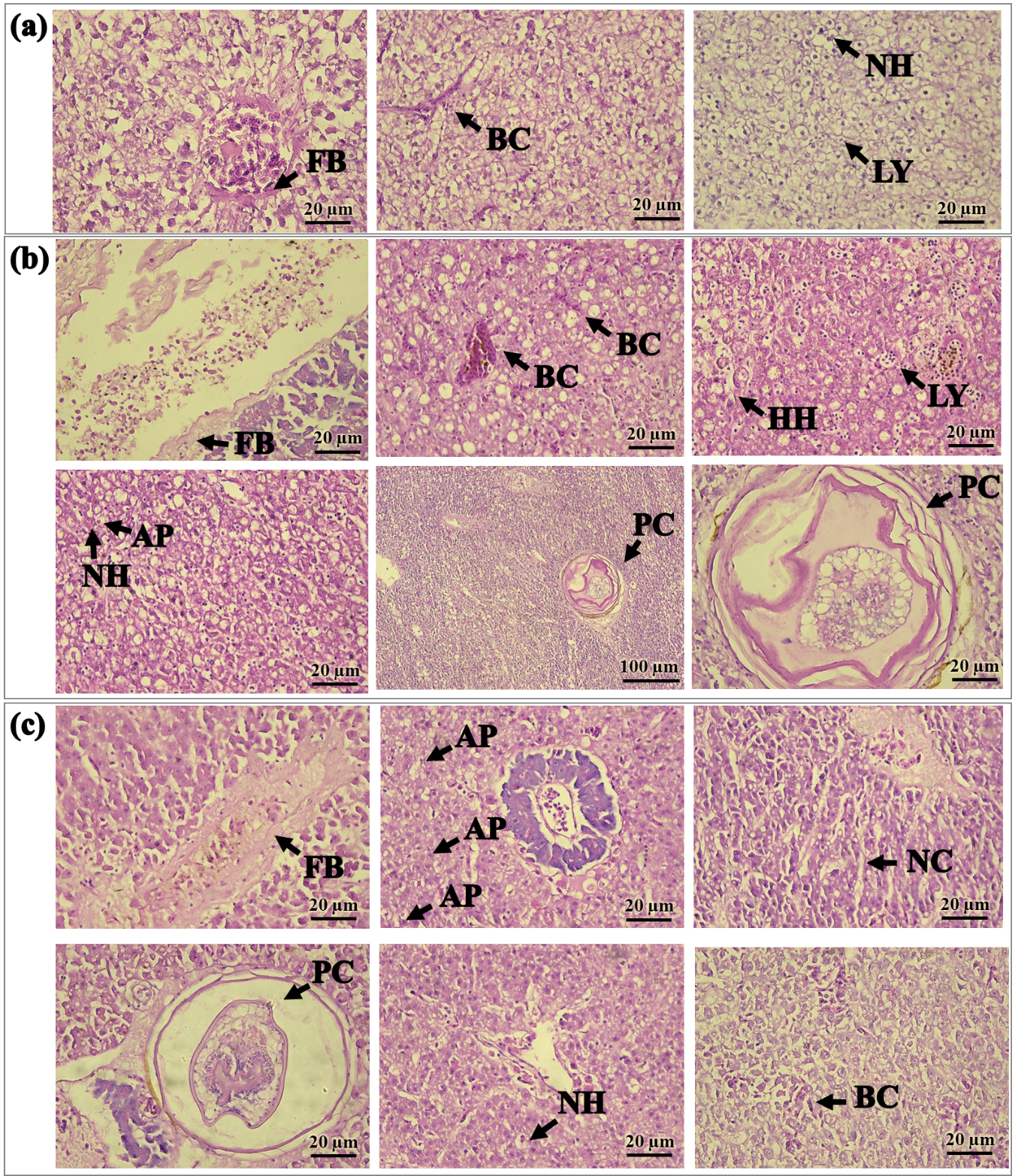
**

**
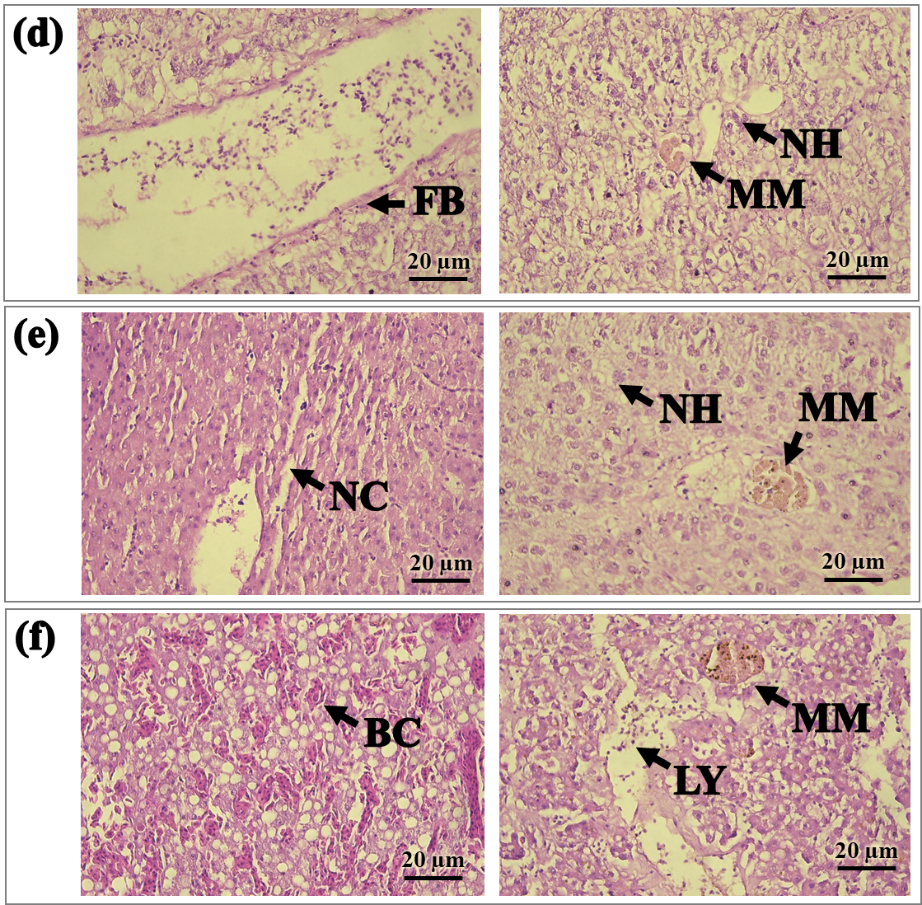
**

**Figure S5.** Histopathology in fish species liver HE40X. **(a)** *H. malabaricus*, **(b)** *P. magdaleniatum*, **(c)** *C. magdalenae*, **(d)** *T. insignis*, **(e)** *C. kraussii*, **(f)** *S. macrurus*.

Abbreviations: Inflammatory lymphocytic infiltrates (LY), necrosis (NC), hepatic congestion (HC), parasite cyst (PC), inflammation with melanomacrophages (MM), nuclear hypertrophy (NH), blood congestion (BC), lipid vacuolization – steatosis (ST), hypertrophy of hepatocytes (HH), apoptosis (AP), and fibrosis of blood vessels with thickened fibrous wall (FB).

**Table S4**. Hazard Quotient (HQ) and maximum allowable rates of fish consumption (CRmw) for adults, pregnant/lactating women, and children aged 3, 4, and 5 years, based on fish from the San Jorge River in San Marcos, La Mojana, Sucre (Colombia).

| **Season** | **Common name** | **Scientific name** | **Adults** | | | **Pregnant/lactating** | | | **Children 3 years olds** | | | **Children 4 years olds** | | | **Children 5 years olds** | | |
| --- | --- | --- | --- | --- | --- | --- | --- | --- | --- | --- | --- | --- | --- | --- | --- | --- | --- |
|  |  |  | EDI | HQ | CRmw | EDI | HQ | CRmw | EDI | HQ | CRmw | EDI | HQ | CRmw | EDI | HQ | CRmw |
| Rainy | Moncholo | *H. malabaricus* | 0.74 | 7.35 | 1 | 0.34 | 3.37 | 2 | 0.52 | 5.21 | 7 | 0.61 | 6.06 | 5 | 0.51 | 5.11 | 5 |
|  | Pacora | *P. surinamensis* | 0.80 | 8.00 | 1 | 0.37 | 3.67 | 2 | 0.57 | 5.67 | 6 | 0.66 | 6.59 | 5 | 0.56 | 5.56 | 4 |
|  | Bagre rayado | *P. magdaleniatum* | 079 | 7.87 | 1 | 0.36 | 3.61 | 2 | 0.56 | 5.57 | 7 | 0.65 | 6.48 | 5 | 0.55 | 5.47 | 4 |
|  | Bagre blanquillo | *S. cuspicaudus* | 0.93 | 9.26 | 1 | 0.42 | 4.24 | 2 | 0.66 | 6.56 | 6 | 0.76 | 7.63 | 4 | 0.64 | 6.43 | 4 |
|  | Chango | *C. magdalenae* | 1.04 | 10.42 | 1 | 0.48 | 4.78 | 1 | 0.74 | 7.39 | 5 | 0.86 | 8.59 | 4 | 0.72 | 7.25 | 3 |
|  | Barbul | *P. virginicus* | 0.68 | 6.75 | 1 | 0.31 | 3.10 | 2 | 0.48 | 4.78 | 8 | 0.56 | 5.56 | 6 | 0.47 | 4.69 | 5 |
|  | Cachegua | *T. insignis* | 0.72 | 7.22 | 1 | 0.33 | 3.31 | 2 | 0.51 | 5.11 | 7 | 0.59 | 5.95 | 5 | 0.50 | 5.02 | 5 |
|  | Mojarra amarilla | *C. kraussii* | 0.98 | 9.82 | 1 | 0.45 | 4.50 | 1 | 0.70 | 6.96 | 5 | 0.81 | 8.09 | 4 | 0.68 | 6.83 | 3 |
|  | Mayupa | *S. macrurus* | 1.37 | 13.71 | 1 | 0.63 | 6.29 | 1 | 0.97 | 9.72 | 4 | 1.13 | 11.30 | 3 | 0.95 | 9.53 | 2 |
| Dry | Moncholo | *H. malabaricus* | 0.61 | 6.13 | 1 | 0.28 | 2.81 | 2 | 0.43 | 4.34 | 8 | 0.50 | 5.05 | 6 | 0.43 | 4.26 | 5 |
|  | Pacora | *P. surinamensis* | 0.54 | 5.44 | 1 | 0.25 | 2.49 | 3 | 0.39 | 3.85 | 9 | 0.45 | 4.48 | 7 | 0.38 | 3.78 | 6 |
|  | Bagre rayado | *P. magdaleniatum* | 0.78 | 7.77 | 1 | 0.36 | 3.56 | 2 | 0.55 | 5.51 | 7 | 0.64 | 6.40 | 5 | 0.54 | 5.40 | 4 |
|  | Bagre blanquillo | *S. cuspicaudus* | 1.25 | 12.52 | 1 | 0.57 | 5.74 | 1 | 0.89 | 8.87 | 4 | 1.03 | 10.31 | 3 | 0.87 | 8.70 | 3 |
|  | Chango | *C. magdalenae* | 1.60 | 16.02 | 0 | 0.73 | 7.34 | 1 | 1.13 | 11.35 | 3 | 1.32 | 13.19 | 2 | 1.11 | 11.13 | 2 |
|  | Barbul | *P. virginicus* | 0.21 | 2.15 | 3 | 0.10 | 0.98 | 7 | 0.15 | 1.52 | 24 | 0.18 | 1.77 | 18 | 0.15 | 1.49 | 16 |
|  | Cachegua | *T. insignis* | 0.83 | 8.29 | 1 | 0.10 | 3.80 | 2 | 0.59 | 5.87 | 6 | 0.68 | 6.83 | 5 | 0.58 | 5.76 | 4 |
|  | Mojarra amarilla | *C. kraussii* | 0.89 | 8.86 | 1 | 0.38 | 4.06 | 2 | 0.63 | 6.28 | 6 | 0.73 | 7.30 | 4 | 0.62 | 6.16 | 4 |
|  | Mayupa | *S. macrurus* | 1.27 | 12.66 | 1 | 0.41 | 5.81 | 1 | 0.90 | 8.97 | 4 | 1.04 | 10.43 | 3 | 0.88 | 8.80 | 3 |

| **HQ<1** | **HQ>1** | **CRmw=1** | **CRmw=0** |
| --- | --- | --- | --- |

Abbreviations: Estimated Daily Intake (EDI), Hazard Quotient (HQ), and Maximum Safe Fish Consumption Rate (meals per week) (CRmw).
